# Modeling of HIV-1 Infection: Insights to the Role of Monocytes/Macrophages, Latently Infected T4 Cells, and HAART Regimes

**DOI:** 10.1371/journal.pone.0046026

**Published:** 2012-09-26

**Authors:** Qiang Li, Furong Lu, Kaifa Wang

**Affiliations:** 1 Department of Device and Equipment, School of Biomedical Engineering and Medical Imaging, Third Military Medical University, Chongqing, People's Republic China; 2 Department of Chemistry, College of Chemistry and Chemical Engineering, Chongqing University, Chongqing, People's Republic China; Fundació Institut d'Investigació en Ciències de la Salut Germans Trias i Pujol. Universitat Autònoma de Barcelona. CIBERES, Spain

## Abstract

A novel dynamic model covering five types of cells and three connected compartments, peripheral blood (PB), lymph nodes (LNs), and the central nervous system (CNS), is here proposed. It is based on assessment of the biological principles underlying the interactions between the human immunodeficiency virus type I (HIV-1) and the human immune system. The simulated results of this model matched the three well-documented phases of HIV-1 infection very closely and successfully described the three stages of LN destruction that occur during HIV-1 infection. The model also showed that LNs are the major location of viral replication, creating a pool of latently infected T4 cells during the latency period. A detailed discussion of the role of monocytes/macrophages is made, and the results indicated that infected monocytes/macrophages could determine the progression of HIV-1 infection. The effects of typical highly active antiretroviral therapy (HAART) drugs on HIV-1 infection were analyzed and the results showed that efficiency of each drug but not the time of the treatment start contributed to the change of the turnover of the disease greatly. An incremental count of latently infected T4 cells was made under therapeutic simulation, and patients were found to fail to respond to HAART therapy in the presence of certain stimuli, such as opportunistic infections. In general, the dynamics of the model qualitatively matched clinical observations very closely, indicating that the model may have benefits in evaluating the efficacy of different drug therapy regimens and in the discovery of new monitoring markers and therapeutic schemes for the treatment of HIV-1 infection.

## Introduction

Human immunodeficiency virus type I (HIV-1) is a lentivirus that causes acquired immunodeficiency syndrome (AIDS), which damages the immune system and leads to life-threatening opportunistic infections [1], [2]. HIV-1 infection and progression and the corresponding immune response have been extensively studied in both biological and mathematical respects.

Studies have shown that the preferred targets of HIV-1 infection are T4 cells and macrophages, despite that wide variety of human cells (hematopoietic cells, brain cells, skin cells, bowel cells, etc.) that are susceptible to HIV-1 infection [3]. When HIV-1 invades a new host, there is an initial burst of viremia, during which plasma RNA levels increase dramatically to about 10^7^ copies/ml. The number of T4 cells drops abruptly. This is in turn followed by an accentuated reduction in the plasma viral load caused by the stimulated immune system [4], [5], [6]. After this stage, there is a prolonged period of clinical latency (i.e., asymptomatic phase), which can last from two weeks to over twenty years but usually lasts about ten years [7]. During this period, there is little detectable viremia, and the concentration of T cells slowly decreases. However, during the asymptomatic phase, viral replication is active in lymph nodes (LNs), which typically become persistently swollen in response to large amounts of viruses that become trapped on the dendritic processes of follicular dendritic cells (FDCs), and about 10^5^ copies/ml of viral RNA molecules are produced daily [5], [6], [8], [9]. When the number of T4 cells declines to below 200 cells/mm^3^, the patient is clinically classified as an AIDS patient. This is characterized by the loss of cell-mediated immunity, the appearance of opportunistic infections, and a rapid increase in the viral load [10]. In this way, HIV-1 infection is typically characterized by three distinct phases: acute infection, asymptomatic phase, and AIDS.

After HIV-1 virions enter the peripheral blood (PB), they have the following three endings. 1) They will be engulfed by monocytes/macrophages and be processed into small peptides. Then certain antigens are expressed on the cell surfaces of these monocytes/macrophages in association with major histocompatibility complex II (MHC-II). Monocytes/macrophages that can present viral antigens to T4 cells are called activated monocytes/macrophages. 2) They infect monocytes/macrophages through coreceptor C-C chemokine receptor type 5 (CCR5), which is expressed on the cell surface. 3) They also infect T4 cells through coreceptor C-X-C chemokine receptor type 4 (CXCR4) or CCR5, which is also expressed on the cell surface. Upon recognition of viral antigens by antigen-presenting activated monocytes/macrophages, naïve T4 cells are activated and become effector cells (helper cells). These helper cells secrete cytokines, that activate B cells and T8 cells, which would become plasma cells and cytotoxic T lymphocytes (CTLs), respectively. Plasma cells secrete HIV-1-specific neutralizing antibodies that can bind to HIV-1 and inactivate it. After binding to the antigens presented by MHC-I on the surfaces of the infected cells, CTLs kill these cells by inducing apoptosis. This is the critical component of HIV-1 control during the acute phase [11]. CTLs can also inhibit viral replication and monocytes/macrophages infection via a non-cytolytic mechanism [12], [13], [14].

Viruses can also seed into LNs in the form of either viral particles or infected monocytes/macrophages. The immune response in the LNs is similar to that observed in PB with the exception that FDCs are involved. FDCs are only found in the B-cell follicles and germinal centers of the peripheral lymphoid tissues. They have the unique capacity to trap pathogens for long periods of time [15] and have been found to trap HIV-1 particles through CD21 receptor [16]. FDCs could maintain their infectivity even in the presence of neutralizing antibodies [15]. The deposition of HIV-1 immune complexes (ICs) in the FDCs network is associated with the concomitant generalized polyclonal activation of B cells, which results in hypergammaglobulinemia and serves as a source of infection for cells that reside in or migrate through the LNs, such as T4 cells [17]. Incremental HIV-1 transcription and viral production in T4 cells in the LNs has also been observed [18].

Because the interaction between HIV-1 and the immune system is complex and dynamic, it is in dynamic and metabolic equilibrium for most of the infection period [19]. This makes the virus difficult to eliminate. In this sense, mathematical models of HIV-1 dynamics can help predict the progression of the infection, and clinical researches combined with mathematical modeling can improve our understanding of HIV-1 infection.

Many mathematical models that mimic the aforementioned immune response to HIV-1 infection have been proposed. Most of these models focus on free viral spread in one compartment (such as PB [20], [21], [22], [23], [24], [25], [26], [27], [28]) and cover no more than two statuses (infected and uninfected) of T4 cells. Some of the models divide the T4 cells by function into memory, activated, and effector cells [20], [25]. Those models mainly focus on one of the three typical stages of HIV-1 infection (acute or latent) [20], [21], [27]. This makes it impossible to predict the comprehensive disease progression. Wasserstein-Robbins [26] focused on T4 cells, T8 cells, and macrophages and gave a relatively detailed description of T4 and T8 cells; the model exhibited typical features of HIV-1 infection. However, in the simulation, the number of T4 cells increased immediately after the viral invasion, exceeding normal levels. This is the exact opposite of clinical observations, which showed that the number of T4 cells decreases abruptly after viral inoculation [29]. It has also been suggested that the viral transmission may only through cell-cell contact during the asymptomatic phase. This is supported by the observation that T4 cells carry only M-tropic HIV-1 variants during this phase of infection [30]. In this way, the Wasserstein-Robbins model cannot resolve certain uncertainties regarding the reaction of T4 cells to HIV-1 infection during the acute and latency periods.

The majority of HIV-1 infections occur in the lymphoid tissues [31], which house 98% of the body's T4 cells [32]. There are three clearly defined stages of LNs destruction during HIV-1 infection: follicular hyperplasia, follicular disruption, and follicular depletion [33]. There is a high level of HIV-1 RNA molecules in the LNs at all stages [34]. In this sense, it is vital to understand the dynamics of infection dynamics within the LNs, to uncover the information regarding cellular infection and viral production, and to describe HIV-1 infection precisely. Several models have discussed those two compartments [35], [36]. The migrations of T4 cells and virus, and the asymptomatic phase of infection are the major factors considered in these models. However, these models do not give detailed descriptions of cell status and ignore the cell-to-cell spread of infection. Most importantly, the results produced by these models are not consistent with the clinical observations of the three stages of LNs destruction observed during HIV-1 infection [33].

HIV-1 is believed to be active in areas such as the brain, and HIV-1 can enter the central nervous system (CNS) soon after peripheral infection of circulating T cells and monocytes [37]. In the Trojan horse model [38], it has been suggested that the virus enters the CNS mainly through infected monocytes/macrophages destined to become brain-resident macrophages or perivascular macrophages. In this way, the cell-cell spread of infection might be much more important in brain than elsewhere, and it has been suggested that HIV-1-associated neurocognitive disorders depend largely on continuous seeding of the CNS with immune-activated leukocytes, mainly monocytes/macrophages from PB [39]. The movement of immune cells and the flow of chemotherapeutic drugs are thought to be restricted by the blood-brain barrier [23]. This makes the CNS being cited as a sanctuary compartment where the viral particles can survive and develop mutants. Therefore, CNS may be an important compartment in HIV-1 infection.

In the present paper, we propose a more realistic model for HIV-1 dynamics. Numerical simulations were performed to determine the various mechanisms underlying the progression of the disease and the immune response under normal infection conditions and the typical highly active antiretroviral therapy (HAART) conditions. The model provides a framework for the integration of multi-faceted information related to HIV-1 infection, including five cell types with various statuses, three distinct compartments, cell-cell spread of T4 cells infection and induction of T4 cell death, and viral evolution. This may increase our understanding of the causes of failure of HAART treatment and may support the development of new drugs.

## Results

In order to assess the validity of the proposed dynamic model and determine its implications for HIV-1 infection, we here performed a numerical simulation using the dynamic model and MATLAB software. This simulation was based on the parameters' values and initial conditions, which will all be described in detail in the Methods Section. The following figures and tables show the results of the simulations. Note that time is expressed in days in some graphs and in years in others. Because long-term experimental and clinical data are difficult to obtain, only the tendencies and qualitative meanings of the results of our simulation are discussed here, although these include changes in the values of the variables.

### Basic model results

#### Three phases of HIV-1 infection and characterization of the stages of lymph nodes destruction

**Figure 1:**
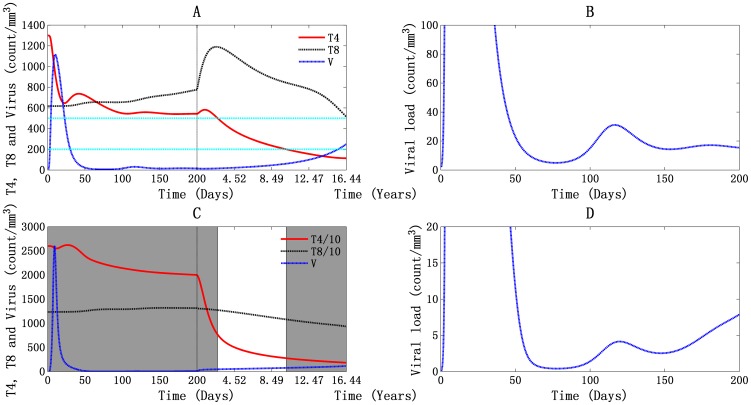
Global simulation of HIV-1 infection dynamic: T4, T8, and viral particles. A and B represent the situation in PB, and C and D represent it in LNs. The double green horizontal lines in panel A represent the T4 cell counts at 500 cells/mm^3^ and 200 cells/mm^3^, which are the time points for LNs stages classification (expressed as shadowed parts for stage 1 and stage 3 in panel C). AIDS occurs at about 3668 days, about 10 years.

A simulation of HIV-1 infection over time considering T4 cells, T8 cells, and viral particles is shown in Figure 1. The dynamics of infected cells throughout the infection process are discussed in Figure S1. The three phases of the infection, acute, chronic, and AIDS, are visible in the graphs. This shows that products of the model match clinical data.

In the PB simulation results shown in Figure 1**A**, after viral inoculation, the number of T4 cells rapidly dropped from the initial value, 1300 cells/mm^3^, to 646 cells/mm^3^, a reduction of about 49.7%. This was because there is at first no adaptive immunity to HIV-1 infection. At the same time, the viral concentration increased, reaching an initial peak of 1116 particles/mm^3^ at 10 days after infection with viral RNA 2.232×10^6^ molecules/ml. When the specific immune response was elicited, the CTL response developed and neutralizing antibodies emerged. Then the viral load dropped abruptly to about 5 viral particles/mm^3^ with 10^4^ RNA molecules/ml at 77 days (Figure 1**B**). Meanwhile, the number of T4 cells gradually increased to about 737 cells/mm^3^ at 42 days after infection, which is about 56.7% of normal T4 cell count at that stage. The number of T4 cells cannot reach the normal level after that [29]. During this period, T8 cell-count increased to 656 cells/mm^3^ and remained relatively unchanged. The second viral peak occurred at 117 days after infection (Figure 1**B**) with 31 viral particles/mm^3^ and 6.2×10^4^ viral RNA molecules/ml. A peak in the T4 cell count accompanied the second viral peak, showing 558 cells/mm^3^ at 131 days. Then the infected cells stimulated a second CTL response, and the T8 cell count increased up to 725 cells/mm^3^. During the asymptomatic phase (Figure 1**A**), the T4 cell count peaked a third time reaching about 582 cells/mm^3^ at 505 days. Then the number decreased gradually, and the viral load remained high. The system descends to AIDS after about 10 years (in 3668 days), when the concentration of T4 cells drops below 200 cells/mm^3^, viral load reaches 61 particles/mm^3^, and viral RNA reaches 1.22×10^5^ molecules/ml (Figure 1**A**).

Clinically, there are three clearly defined stages of LNs destruction in HIV-1 infection: follicular hyperplasia (associated with T4 cell counts above 500 cells/mm^3^ in PB); follicular disruption ( associated with T4 cell counts of 200—500 cells/mm^3^ in PB), and follicular depletion [33], [34]. During the first stage, LNs appear to be normal reactive nodes and associate with the hyperplastic germinal centers. Next, the germinal centers are gradually lost, the LNs thicken, and small blood vessels become irregularly distributed. This is called follicular disruption. Finally, with the depletion of lymphocytes and the shrinking of the LNs, the LNs architecture is completely destroyed [33], [40].Our model simulated the characteristics of the different stages reasonably well (Figures 1**C and D**).

During the first stage (from day 0 to day 1004), shown in Figure 1**C**, after a slight decrease about 50 cells/mm^3^, the T4 cell count increased to 26,210 cells/mm^3^ within 27 days, which is even higher than normal levels. This is quite different from what happened in the PB, where there is at first a dramatic decrease in T4 cell count. This is because, during the first viremia, HIV-1 RNA is confined within the germinal centers, where it is attached to FDCs via molecules called C-type lectin [33]. In this area, there are relatively few T4 cells, only about 5% of total T4 cells. When the HIV-1-specific macrophages become active, cell-mediated immunity begins. This is accompanied by a significant proliferation and differentiation of T4 cells. The first viral peak occurred at 10 days, with 2607 viral particles/mm^3^ and 5.214×10^6^ viral RNA molecules/ml. As the CTL response emerged and neutralizing antibodies were produced, the viral load dropped dramatically to less than 1 viral particle/mm^3^ and 800 RNA molecules/ml at 78 days after infection. A second peak formed at 120 days, with 4 viral particles/mm^3^ and 8×10^3^ RNA molecules/ml (Figure 1**D**). It has been shown that HIV-1-induced follicular expansion in this stage is characterized by the predominant expansion of T8 cells [33]. This is shown in our simulation results. As shown in Figure 1**C**, the T4 cell count dropped from 25,998 cells/mm^3^ to about 7645 cells/mm^3^, but the T8 cell count, increased from 12,349 cells/mm^3^ to 12,731 cells/mm^3^.

During the second stage (Figure 1**C**), which lasted from day 1004 to day 3668, the T4 cell level continues to fall, dropping to about 2774 cells/mm^3^ in the end. Meanwhile, the T8 cell count dropped to about 10,802 cells/mm^3^. The viral load increased to about 76 viral particles/mm^3^ with 1.52×10^5^ RNA molecules/ml. All these results are characteristic of follicular disruption.

After 3668 days (which means the patient is at AIDS), the cell counts of T4 and T8 cells kept decreasing while the viral load kept rising, and the LNs are in the follicular depletion stage (Figure 1**C**).

#### Inversion of T8/T4 ratio with a bottle-neck value and T8 cell hyper-activation

**Figure 2:**
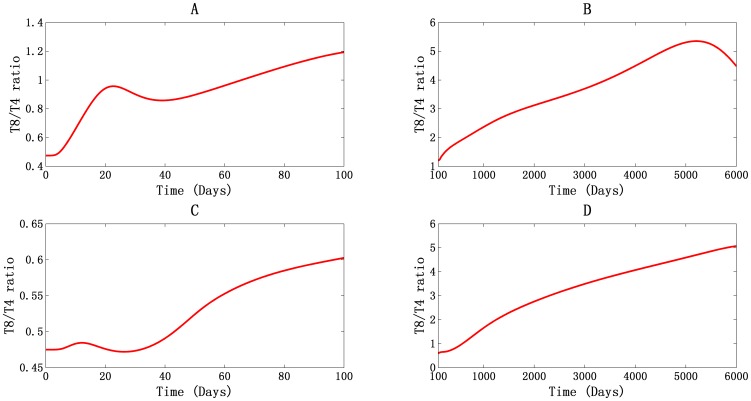
The T8/T4 ratio. Changes in the T8/T4 ratio change in PB (A and B) and LNs (C and D), during the first 100 days (A and C) and from day 100 to day 6000 (B and D).

The T8/T4 ratio, shown in Figure 2, was initially 0.475 for both PB (Figure 2**A**) and LNs (Figure 2**C**). This ratio became inverted after 65 days for PB (Figure 2**A**) and 578 days for LNs (Figure 2**D**). The pattern of inversion was different in the two different compartments. In PB (Figure 2**A**), the ratio increased at first, reaching about 0.958 at 22 days after infection. It then fell to about 0.86 in 38 days. After that, it continued to rise, reaching a peak value of about 5.392 at 5210 days (Figure 2**B**). It then decreased again. In LNs, there was only a slight increase of the ratio, to about 0.484 at 11 days after infection. It then fell to about 0.472 at 26 days, which is below pre-infection levels (Figure 2**C**). After that, the ratio increased continuously, showing no peak value at any point during our 6000-day simulation. The different patterns of change in the T8/T4 ratio in those two compartments are the results of different T4 cells dynamics during the first viremia: In PB, the T4 cell count dropped abruptly during the first viremia. In the LNs, there was only a slight decrease, after which the cell count increased to levels above normal. In addition, the peak value in PB and a tendency of forming peak value in LNs suggested that the inverted ratio would not keep increasing. This is because the activation and proliferation of naïve T8 cells requires help from T4 cells and activated macrophages, whose functional activities are reduced during HIV-1 infection. For this reason, the ratio of T8/T4 can be expected to bottleneck.

**Figure 3:**
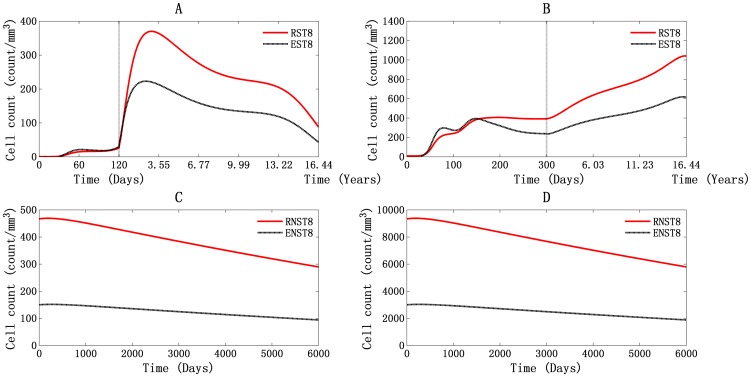
Dynamics of T8 cells in PB and LNs. Panels A and C stand for PB, and panels B and D stand for LNs.

A numerical simulation of T8 cell dynamics in PB and LNs is shown in Figure 3. Figures 3**A and 3B** show the dynamics of HIV-1-specific T8 cells (ST8) during infection, including resting ST8 (RST8) and effector ST8 (EST8) in both compartments. Figures 3**C and 3D** show the characteristics of non-specific T8 cells (NST8) in each compartment. After HIV-1 invasion, RST8 recognized the antigens presented by the activated macrophages. With the help of HIV-1-specific effector T4 cells, they underwent proliferation and differentiated into EST8 (Figure 3**A** for PB and Figure 3**B** for LNs). The HIV-1-specific CTL response, which is characterized by the proliferation of EST8, started at 9 days for PB and at 16 days for LNs (Figures 3**A and 3B**). EST8 proliferation began around the time of the first viremia, followed by a dramatic decline in viral load. This emphasizes the critical role of CTL response in controlling HIV-1 infection. There were two peaks for EST8 in both PB and LNs, about 21 cells/mm^3^ at 64 days and 223 cells/mm^3^ at 900 days in PB and about 298 cells/mm^3^ at 79 days and 394 cells/mm^3^ at 148 days in LNs (Figures 3**A and 3B**). The double peaks of EST8 in LNs were closely correlated with the wave hollows of viral load (Figure 1**D**), and the peaks in PB did not show the same correlation with viral peaks but rather showed a delay. This phenomenon was qualitatively correlated with the actual immune response. When pathogens invade human body, the antigen-presenting cells phagocytize the pathogens and migrate into the lymph nodes, where they activate cell-mediated immunity. Then, the activated cells efflux from the LNs into the PB and function as immune cells to control the infection. After the acute infection, the cell counts of EST8 in PB and LNs both remained relatively high, with values of about 43 cells/mm^3^ and 615 cells/mm^3^, respectively, at 6000 days (Figures 3**A and 3B**). Regarding NST8 dynamics, the numbers decreased gradually during the simulation, reaching about 94 cells/mm^3^ in PB and about 1886 cells/mm^3^ in LNs at 6000 days (Figures 3**C and 3D**).

The results of our simulation demonstrate that T8 cells are in a hyper-activation status during the HIV-1 infection [41], [42], [43]. This means that the HIV-1-specific T8 cell count remains higher than normal levels, especially in LNs, in which the number continued to grow even during the AIDS phase (Figure 3**B**). The tendency to reach peak cell count can be seen in Figure 3**B**, as can the decrease in the non-specific T8 cell count. This confirms our previous hypothesis that the T8/T4 ratio would peak in LNs. It has been shown that the hyper-activated T8 cells colocalize with HIV-1 infected cells but no sustained reduction in those infected cells has been observed in clinical settings [44]. Only a few of these T8 cells remain able to suppress viral replication [45]. All of these previous findings match the results of our simulation. It has also been suggested that a lack of adequate help from T-cell, changes in antigen presentation by macrophages, and reduced recognition of HIV-1-infected cells as a result of Nef down-regulates MHC-I molecules on the cells surface [46], may all account for the impaired CTLs function [47], [48].

#### Triple T4 cell peaks during the HIV-1 infection, reaction to the viral invasion and self-replenishment

**Figure 4:**
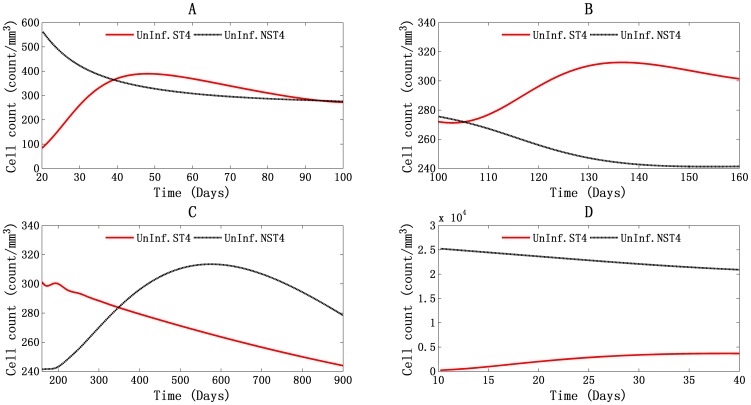
Illustration of uninfected HIV-1-specific and non-specific T4 cells during the global simulation results in PB and LNs. Panels A, B and C stand for PB, and panel D stands for LNs. The T4 cell count peaks in PB three times: (A) from day 20 to day 100, (B) from day 100 to day 160, and (C) from day 160 to day 900.

In Figure 1**A** of the global simulation of PB, three peaks of T4 cell count are observed. The first two peaks were correlated with the double viral peaks, which began at 22 days and 107 days and reached their peak values of 737 cells/mm^3^ and 558 cells/mm^3^ at 42 days and 131 days, respectively. As shown in Figures 4**A and 4B**, the proliferation of uninfected HIV-1-specific T4 cells (ST4) accounts for those two peaks, with about 265 cells/mm^3^ (114 cells/mm^3^ at 22 days and 379 cells/mm^3^ at 42 days) and 37 cells/mm^3^ (273 cells/mm^3^ at 107 days and 310 cells/mm^3^ at 131 days) increased. The uninfected HIV-1 nonspecific T4 cells (NST4) appeared to act in an opposite way. Their cell count fell from 527 cells/mm^3^ to 352 cells/mm^3^, and from 270 cells/mm^3^ to 246 cells/mm^3^ in the same day. The third peak began on day 205 at a cell concentration of 543 cells/mm^3^ and reached its peak of 582 cells/mm^3^ on day 505. From Figure 4**C**, we can see that the proliferation of uninfected HIV-1 nonspecific T4 cells accounted for this peak, with an increase of about 66 cells/mm^3^ (244 cells/mm^3^ on day 205 and 310 cells/mm^3^ on day 505). Uninfected HIV-1-specific T4 cells acted in an opposite way, showing a decrease of about 29 cells/mm^3^ (299 cells/mm^3^ on day 205 and of 270 cells/mm^3^ on day 505).

These results show that the first two cell-count peaks represent the stimulation of the immune system by the invading HIV-1 virions. The magnitude of the immune response is proportional to the viral load, as shown in the T4 cells proliferation count. The third peak represents the recovery of the total T4 cells count, capturing the clinical observation that HIV-1-specific T4 cell proliferation is insufficient or might be absent after acute infection [49], [50]. These results show that after acute infection phase, although the T4 cell count recovered to some degree, the true functional T4 cell count, the number of HIV-1-specific T4 cells, continued to fall. This may represent the gradually loss of body's ability to control HIV-1 infection.

In the LNs, as shown in Figure 1**C**, only one T4 cell count peak was observed. It is attributable to the proliferation of uninfected HIV-1-specific T4 cells (Figure 4**D**). This is the response of the immune system to invading HIV-1 in LNs. However, the T4 cell count did not recover at any point during the infection period. There was a massive depletion of T4 cells in LNs, indicates that the LNs are the major location of T4 cell death.

#### T4 cell depletion due to the death of uninfected T4 cells and the role of infected macrophages

**Figure 5:**
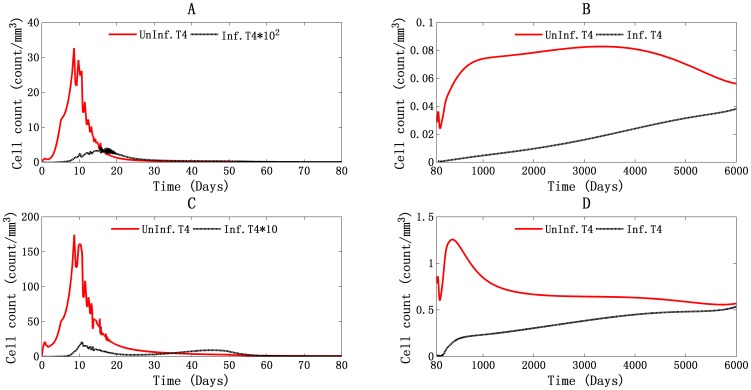
Daily death of T4 cells in PB and LNs. The daily death of infected and uninfected T4 cells in PB (A and B) and LNs (C and D), during the first 80 days (A and C) and from day 80 to day 6000 (B and D).

Figure 5 shows the daily death rate of infected and uninfected T4 cells in PB and LNs. The graphs demonstrate that the uninfected T4 cells are the major type of cell to die during the infection process, especially during acute infection, during which the death rate of these cells is about two orders of magnitude greater than that of infected T4 cells in PB (Figures 5**A and 5B**), and one order of magnitude greater in LNs (Figures 5**C and 5D**). These results support the observations that apoptosis occurs predominantly in uninfected T4 cells and that fewer apoptotic cells are physically infected by virus [51].

And we recognize that it is vital to make a precise description of T4 cell death with the following considerations. Firstly, it has been suggested that the indirect mechanisms of HIV-1-associated apoptosis may play a major role in infection, especially in the apoptosis of uninfected T4 cells [51]. Macrophage-mediated cell death appears to be selective for uninfected T4 cells, and macrophage-mediated T4 cell apoptosis has implications in vivo in that levels of tissue apoptosis are directly correlated with levels of macrophage-associated FasL [52], [53]. This suggests that macrophages play a major role in this indirect mechanism.

**Figure 6:**
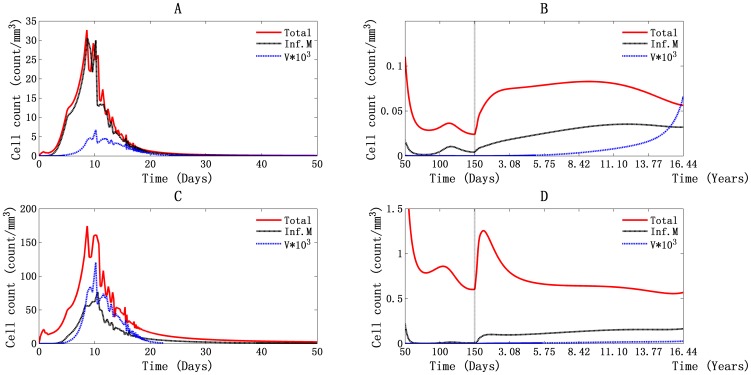
Illustration of the factors causing T4 cells death. Effects of macrophage-mediated, virus-mediated, and total uninfected T cell death in PB (A and B) and LNs (C and D), during the first 50 days (A and C) and from day 50 to day 6000 (B and D).

In order to determine the major factor that impacts T4 cell death, we compared the effects of macrophage-mediated, virus-mediated, and total uninfected T4 cell death in PB and LNs (Figure 6). Macrophage-mediated death of uninfected T4 cells accounted for the greatest number of deaths of uninfected T4 cells in both compartments. This supports the clinical observations described in previous studies [54], [55]. The first peak occurred about 8 days after infection (Figures 6**A and 6C**), when the total number of deaths of uninfected T4 cells was about 32.7 cells/mm^3^ per day in PB and about 175.3 cells/mm^3^ per day in LNs. Macrophage-mediated cell deaths was about 30.5 cells/mm^3^ per day and 76.9 cells/mm^3^ per day, accounting for 93.3% and 43.9% of the total number of deaths of T4 cells. Virus-mediated T4 cell deaths only numbered about 6.78×10^−3^ cells/mm^3^ per day and 0.12 cells/mm^3^ per day, respectively. With the second viremia (Figures 6**B and 6D**), which occurred at 116 days after infection, the numbers reached about 0.037 cells/mm^3^ per day, 0.01 cells/mm^3^ per day, and 1.38×10^−7^ cells/mm^3^ per day in PB and about 0.87 cells/mm^3^ per day, 0.17 cells/mm^3^ per day, and 4×10^−8^ cells/mm^3^ per day in LNs. The simulated results of daily T4 cell deaths showed a third peak in LNs at 435 days after infection (Figure S2). Although macrophage-mediated T4 cell death was the major cause of T4 cell death, this peak was caused primarily by the increased self-induced death among T4 cells (Figure 6**D**).

#### Persistent production of virus mainly by infected monocytes/macrophages and the contributions of virus-mediated and cell-cell spread of T4 cells infection

**Figure 7:**
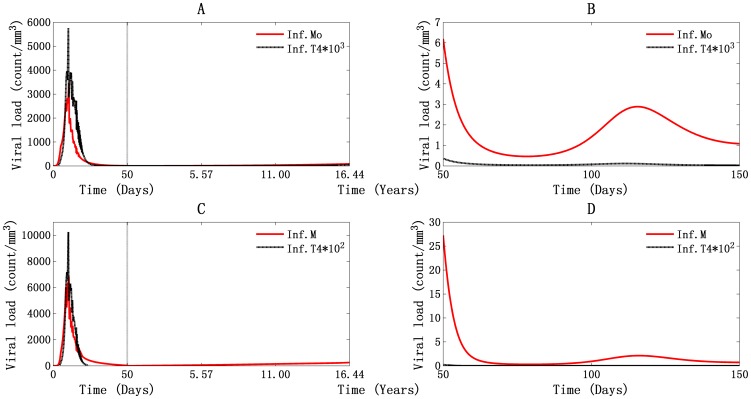
Illustration of viral production by infected monocytes/macrophages and actively infected T4 cells. Panels A and B stand for PB, and panels C and D stand for LNs. The second viral peak lasted from day 50 to day 150 (B and D).

A comparison of daily viral production by infected monocytes/macrophages and actively infected T4 cells is shown in Figure 7 for PB and LNs. Our simulations demonstrate that the major virus-producing cells in both PB and LNs are infected monocytes/macrophages throughout the HIV-1 infection process. Viral production by infected monocytes/macrophages was found to be responsible for the double viral peaks.

In PB, daily viral production first peaked at about 3.44×10^3^ particles/mm^3^ with 6.88×10^6^ viral RNA molecules/ml (Figure 7**A**). This supports the experimental implication that during the first viremia, plasma RNA levels are approaching 10^7^ copies/ml [4]. The number was about 6.94×10^3^ particles/mm^3^ with 1.39×10^7^ RNA molecules/ml in LNs (Figure 7**C**). Because the volumes of PB and lymphoid tissues were assumed to be about 5 L and 11 L for each, the total number of viral RNA molecules reached about 1.87×10^11^ in the entire body. At the second viral peak, the values reached 3 particles/mm^3^ in PB (Figure 7**B**), 2 particles/mm^3^ in LNs (Figure 7**D**), and in total about 7.4×10^7^ RNA molecules in the body. This was a decline of four orders magnitude relative to the first viral peak.

During the asymptomatic phase, daily production of virus was about 15 particles/mm^3^ with viral RNA 3×10^4^ molecules/ml in PB (Figure 7**A**) and 50 particles/mm^3^ with viral RNA 10^5^ molecules/ml in LNs (Figure 7**C**). This supports the observation that, during the latency period, daily production of viral RNA molecules in LNs is about 10^5^ copies/ml and about 1.25×10^9^ RNA molecules in the human body [9]. When the T4 cell count fell below 200 cells/mm^3^, the daily production of virus was about 27 particles/mm^3^ and 128 particles/mm^3^ in PB and LNs, respectively. In this way, total viral production was about 3.1×10^9^ RNA molecules in the whole body (Figures 7**A and 7C**), which supports the observation that the viral concentration begins to rise rapidly during the AIDS phase [10].

**Figure 8:**
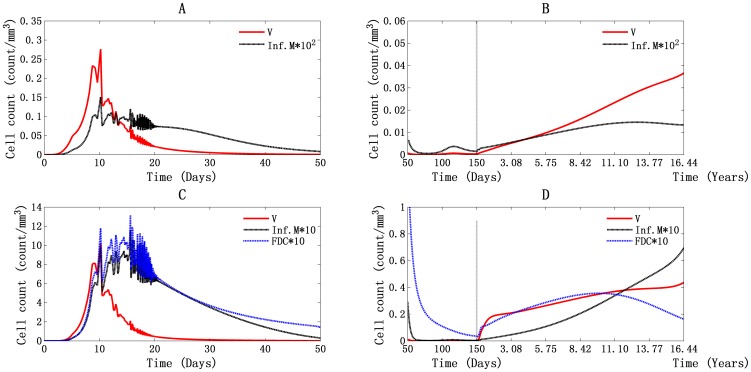
Illustration of T4 cell infection in PB and LNs. T4 cell infection caused by free virus, infected macrophages, and FDC in PB (A and B) and LNs (C and D), during the first 50 days (A and C) and from day 50 to day 6000 (B and D).

The T4 cell infection mediated by free virus, infected macrophages, and FDC in PB and LNs is shown in Figure 8. In Figures 8**A and 8C**, we can see that T4 cell infection during the first viremia was mainly caused by free virus in both PB and LNs. The top values reached about 0.275 cells/mm^3^ per day and 10.18 cells/mm^3^ per day. This is about two orders of magnitude greater and one order greater than the numbers mediated by infected macrophages, respectively. After that, although the virus was still the main cause of T4 cell infection in PB, FDCs were more in LNs, about 7×10^−3^ cells/mm^3^ per day at 119 days, and the number of virus-mediated infections was about 3.6×10^−3^ cells/mm^3^ per day at the same time (Figure 8**D**). When the system goes into latent period and throughout the rest time, free virus still dominates the infection of T4 cells, both in PB and LNs (Figures 8**B and 8D**). As shown in Figure 8**D**, the portion of T4 cell infection caused by FDCs continued to rise throughout the asymptomatic phase, reaching its peak value of about 0.036 cells/mm^3^ per day in 3630 days, which is around the time of onset of the AIDS phase (3668 days).

HIV-1 can spread between cells either through cell-virus or cell-cell transfer, and the mode of cell-cell contact is likely to play a major role in viral dissemination in crowded LNs, where cell contacts are frequent [56], [57]. According to our simulation, although free virus was still the main cause of T4 cell infection in both PB and LNs, an increasing amount of cell-cell spread of infection could be seen in LNs, especially during the second viremia, in which FDC^i^ accounted for the largest number of T4 cell infections. During parameter estimation, a small fraction of infections was assumed to take place when infected macrophages came into contact with T4 cells. Only HIV-1-specific T4 cells can be infected in this way. This reduces the portion of total T4 cell infections that can be attributed to cell-cell interactions.

#### Depletion of infected monocytes/macrophages, control of viral load, and T4 cell count

As indicated by the simulation results of activated and infected monocytes/macrophages (Figure S3), monocytes/macrophages dynamics was closely correlated to the viral dynamics and the disease progression. This suggests that monocytes/macrophages may be suitable as markers for monitoring patients. We have shown that infected monocytes/macrophages play an important role in inducing T4 cell infection and apoptosis and their massive contribution to viral production. The results of the present simulation indicate that monocytes/macrophages play a role in the HIV-1 infection process. Their effects are illustrated in the following graphs.

**Figure 9:**
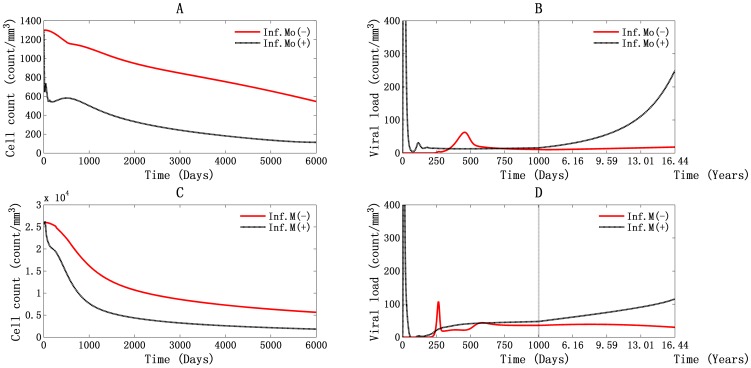
Effects of infected monocytes/macrophages on the T4 cell count and viral load. Panels A and B stand for PB, and panels C and D stand for LNs.

The role of infected monocytes/macrophages in HIV-1 infection mainly contains three parts: induction of T4 cell infection, apoptosis, and production of viral particles. We may therefore evaluate their effects by comparing the change in T4 cell count and viral load under different states. In the present study, we simulate the effects of infected monocytes/macrophages by setting the infection rate of healthy monocytes/macrophages to zero. The changing patterns of T4 cells and virus dynamics in PB and LNs are shown in Figure 9.

In the simulation of T4 cell dynamics, a significant increase appeared in total T4 cell count when we deleted the influence of infected monocytes/macrophages. This took place in both PB and LNs (Figures 9**A and 9C**). With the infected monocytes/macrophages, the time necessary to develop AIDS was about 3668 days (Figure 1**A**). At that point, without monocytes/macrophages, the T4 cell count was 785 cells/mm^3^ in PB (Figure 9**A**), about four times greater than that with those cells. In LNs, without the infected monocytes/macrophages, the T4 cell count was 7661 cells/mm^3^ at 3668 days, about three times greater than with the infected monocytes/macrophages (Figure 9**C**). In the aforementioned (Figure 1A), HIV-1 infection was accompanied by a significant decrease in T4 cell count at the first viremia, and the cell count dropped from its initial value of 1300 cells/mm^3^ to 646 cells/mm^3^ with about 49.7% decreased in PB. When the influence of infected monocytes/macrophages was removed, only a slight decrease in T4 cell count could be seen. It was about 1160 cells/mm^3^ at 560 days, constituting a decrease of only about 10.8% in PB (Figure 9**A**). The slower decrease in T4 cell count has also been seen in LNs (Figure 9**C**). Without the infected monocytes/macrophages, the total T4 cell count at 6000 days was about 545 cells/mm^3^ and 5647 cells/mm^3^ in PB and LNs, respectively (Figures 9**A and 9C**). This is about five times and three times greater than with infected monocytes/macrophages, by about 114 cells/mm^3^ and 1839 cells/mm^3^ (Figures 1**A and 1C**).

The dynamics of T4 cells are associated with changes in viral loading. As shown in Figure 9**B**, only one viral peak appeared in PB when the effects of infected monocytes/macrophages were removed. However, there were still two viral peaks in LNs (Figure 9**D**). The time course for the first viral peak was delayed to about day 457 in PB (Figure 9**B**) and to about day 264 in LNs (Figure 9**D**). The second viral peak in LNs occurred 585 days after infection (Figure 9**D**). The peak values of first viremia were also decreased, showing 62 particles/mm^3^ and 1.24×10^5^ RNA molecules/ml in PB (Figure 9**B**) and about 107 particles/mm^3^ with 2.14×10^5^ RNA molecules/ml in LNs (Figure 9**D**). In situations in which infected monocytes/macrophages were included, 1116 particles/mm^3^ and 2.232×10^6^ RNA molecules/ml and 2607 particles/mm^3^ with 5.214×10^6^ RNA molecules/ml were observed for each part (Figure **1**). Unlike the first viral peak, the second peak value in LNs, which was about 4 viral particles/mm^3^ with 8×10^3^ RNA molecules/ml in natural infection (Figure 1**D**), becomes larger when infected monocytes/macrophages are removed, to be 43 particles/mm^3^ with 8.6×10^4^ RNA molecules/ml (Figure 9**D**). Throughout the simulation, a relatively stable viral load was observed in both compartments when the effects of infected monocytes/macrophages were removed (Figures 9**B and 9D**), but the viral load increased quickly if those infected cells were included.

From these results, we can conclude that infected monocytes/macrophages may determine the progression of the disease and that they are the major cause of T4 cell death and viral production. When their effects are removed, continuous control of viral load can be observed. This may expand patient life expectancy. In this way, monocytes/macrophages may be the most important type of cell in the progression of HIV-1 infection and must not be neglected in the study of both mathematical modeling and biological mechanisms.

### Sensitivity analysis

In order to make a sensitivity analysis of our model using different values of estimated parameters and initial conditions, we mimicked a perturbed system under wide range of individual responses to HIV-1 infection with values varying by ±10%. The results are summarized in Table 1.

**Table 1:** Simulation of ±10% single initial condition/parameter perturbations.

Initial condition/Parameter	Variation	Double viral peaks	Time until AIDS	Infected T4 count and total T4 count on day 6000	Viral load on day 6000
Age	−10%	1116/31 (2606/4)	3789	43/118 (153/1998)	249 (114)
	0%	1116/31 (2607/4)	3668	40/114 (144/1839)	250 (115)
	+10%	1116/31 (2607/4)	3546	37/109 (135/1691)	250 (116)
T4 cell count	−10%	1121/30 (2668/4)	3668	40/114 (144/1839)	250 (115)
	0%	1116/31 (2607/4)	3668	40/114 (144/1839)	250 (115)
	+10%	1111/31 (2544/4)	3668	40/114 (144/1839)	250 (115)
T8 cell count	−10%	1116/31 (2607/4)	3668	40/114 (144/1839)	250 (115)
	0%	1116/31 (2607/4)	3668	40/114 (144/1839)	250 (115)
	+10%	1116/31 (2606/4)	3668	40/114 (144/1839)	250 (115)
Viral particle count	−10%	1116/31 (2600/4)	3668	40/114 (144/1839)	250 (115)
	0%	1116/31 (2607/4)	3668	40/114 (144/1839)	250 (115)
	+10%	1116/31 (2604/4)	3668	40/114 (144/1839)	250 (115)
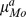 / 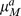	−10%	1108/30 (2578/2)	3659	37/110 (58/1935)	248 (86)
	0%	1116/31 (2607/4)	3668	40/114 (144/1839)	250 (115)
	+10%	1124/31 (2623/5)	3679	43/117 (785/2334)	251 (152)
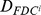	−10%	1116/31 (2607/4)	3664	41/114 (34/2269)	250 (62)
	0%	1116/31 (2607/4)	3668	40/114 (144/1839)	250 (115)
	+10%	1116/31 (2607/4)	3672	39/113 (941/2407)	249 (163)
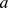	−10%	1083/44 (2232/8)	3877	47/150 (140/2011)	157 (95)
	0%	1116/31 (2607/4)	3668	40/114 (144/1839)	250 (115)
	+10%	1154/25 (3089/3)	3426	93/100 (3559/4645)	957 (292)
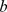	−10%	1116/31 (2607/4)	3669	40/114 (144/1839)	250 (115)
	0%	1116/31 (2607/4)	3668	40/114 (144/1839)	250 (115)
	+10%	1116/31 (2607/4)	3668	40/114 (144/1839)	250 (115)
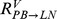	−10%	1126/31 (2601/4)	3673	39/113 (149/1839)	249 (116)
	0%	1116/31 (2607/4)	3668	40/114 (144/1839)	250 (115)
	+10%	1106/31 (2603/4)	3663	41/114 (139/1840)	250 (114)
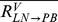	−10%	1109/31 (2610/4)	3664	40/114 (140/1839)	250 (114)
	0%	1116/31 (2607/4)	3668	40/114 (144/1839)	250 (115)
	+10%	1123/31 (2596/4)	3672	39/113 (148/1839)	249 (116)
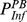	−10%	1116/31 (2607/4)	3649	39/112 (133/1838)	253 (113)
	0%	1116/31 (2607/4)	3668	40/114 (144/1839)	250 (115)
	+10%	1116/31 (2607/4)	3688	40/115 (156/1842)	246 (117)
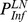	−10%	1116/31 (2602/3)	3662	41/114 (44/2119)	250 (72)
	0%	1116/31 (2607/4)	3668	40/114 (144/1839)	250 (115)
	+10%	1116/31 (2604/5)	3675	38/113 (1623/3062)	249 (169)
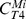	−10%	1116/31 (2606/4)	3667	40/114 (91/1937)	250 (97)
	0%	1116/31 (2607/4)	3668	40/114 (144/1839)	250 (115)
	+10%	1116/31 (2607/4)	3669	40/113 (273/1830)	250 (137)
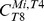	−10%	1116/31 (2607/4)	3668	40/114 (145/1839)	250 (116)
	0%	1116/31 (2607/4)	3668	40/114 (144/1839)	250 (115)
	+10%	1116/31 (2606/3)	3668	40/114 (142/1839)	250 (115)
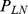	−10%	1114/31 (2524/5)	3669	39/113 (635/2217)	249 (143)
	0%	1116/31 (2607/4)	3668	40/114 (144/1839)	250 (115)
	+10%	1117/31 (2679/3)	3667	40/114 (56/1914)	250 (88)
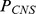	−10%	1117/31 (2625/3)	3668	40/114 (104/1846)	249 (106)
	0%	1116/31 (2607/4)	3668	40/114 (144/1839)	250 (115)
	+10%	1115/31 (2575/4)	3668	40/114 (210/1862)	250 (125)
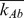	−10%	1121/31 (2658/3)	3685	38/112 (908/2377)	248 (163)
	0%	1116/31 (2607/4)	3668	40/114 (144/1839)	250 (115)
	+10%	1112/31 (2553/4)	3661	40/114 (968/2432)	250 (163)

Note: The numbers in parentheses represent the results in LNs and those not in parenthese are results in PB.

The results show that our model is robust for all ±10% single-parameter perturbations. The time elapsing before the development of AIDS increased from 3426 days to 3877 days, but it remained around 3665 days for most perturbations. With a ±10% change in initial conditions, the time required to develop AIDS changed from 3546 days to 3789 days. Although there were more T4 and T8 cells and a relatively strong immune response during acute infection, viral inoculation load appeared to be an important determinant of disease outcome.

The most significant change in the disease outcomes, as indicated by the time before the onset of the AIDS phase, was the parameter *a*, which represents the influence of virus on the proliferation of uninfected T4 cells. The time required for the development of AIDS ranged from 3877 days to 3426 days when *a* was allowed to vary by 10% in each direction. It was increased by about 451 days when inhibition increased. The effects of viral inhibition on the proliferation of uninfected T4 cells accompanied by the increases in viral peaks during acute infection, increased the ratio of infected T4 cells to total T4 cells at 6000 days and significantly increased of viral load at 6000 days. This confirmed the clinical observation that the proliferation of HIV-1-specific T4 cells is inversely correlated to viral load and can be lost during acute infection. It may be possible that they persist but are anergic during chronic infection, compromising the immune response, causing the AIDS phase to begin earlier.

### Numerical results of HAART-treated model

The effects of the timing of the initiation of antiretroviral therapy have been a controversial part of the evaluation of the benefits of therapy and of its associated short- and long-term complications [58], [59]. In order to simulate the differences in the dynamics of HIV-1 infection between different times of onset of therapy, the times of beginning treated time (BTT) were assumed as following: on the day of infection, on day 1000, day 2000, day 3000, and day 4000. Because the clinical benefits of HAART are strongly correlated with CD4^+^ cell recovery [60], [61], and because the baseline T4 cell count remains the most relevant predictor of clinical progression and survival in patients on HAART [62], we mainly concern the T4 cell count and the viral load under antiviral treatment. The results show that there is a sharp response to drug treatment characterized by an increase in the number of T4 cells and a decrease in viral load.

The following tables show the main results of the simulations. Only partial results are listed in this section. The rest, which are not listed here, characterize the same features.

### Higher drug efficiency, better outcomes, characterized by the viral load and T4 cell count, accompanied by a decrease in Inf.T4/T4 ratio

The effects of the drug efficiency are given in Table 2. Fixed values of drugs effectiveness are shown in different cells and compartments. The results show that more efficient antiviral drugs can produce a dramatic decline in viral peaks during the acute infection. A decreased viral load was observed at 6000 days, accompanied by a significant increase of total T4 cell count in both PB and LNs. However, the impacts of drugs on infected T4 cell count were the opposite (Table 2, fifth column). The number of infected T4 cells was increasing, accompanied by the increased total T4 cell count, both in PB and LNs, although there are exceptions in PB when drug efficiency is 100%. Fortunately, when we calculate the ratio of infected T4 cell count to total T4 cell count in 6000 days, some interesting results were uncovered. As shown in the last column in Table 2, accompanying by the increase of drug efficiency, the portion of infected T4 cells decreases both in both PB and LNs. If we compare the post-therapy ratio to the pre-therapy ratio, a slight decrease in the percentage of infected T4 cells in PB can be seen, but a significant increase in the ratio can be seen in LNs.

**Table 2:** Simulation of different levels of drug efficiency.

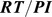	Double viral peaks	Viral load on day 6000	T4 cell count on day 6000	Infected T4 cell count on day 6000	Inf. T4/total T4
0	1116/5(2607/4)	250(115)	114(1839)	40(144)	35.1%(7.8%)
0.25	565/23(1774/3)	44(86)	343(4759)	127(2647)	37.0%(55.6%)
0.5	206/15(1026/*)	23(61)	520(5623)	149(2868)	28.7%(51.0%)
0.75	44/20(612/20)	17(45)	687(6508)	187(2921)	27.2%(44.9%)
1	10/<1(379/26)	2(33)	838(7864)	55(3072)	6.6%(39.1%)

Note: The numbers in parentheses are the results in LNs and those not in parentheses are the results in PB.

* indicates that the second peak was absent. Here 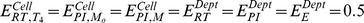, and BTT is day 0.

### With less inter-cell drug effectiveness differences, infected T4 cell count decreases

We also simulated the influence of the effectiveness of different drugs on different types of cells and compartments during the development of the disease. The results are given in Tables 3 and 4. In Table 3, as expected, improved control of viral load, an increase in the total T4 cell count at 6000 days, an increase in the infected T4 cell count on day 6000, and a decreased percentage of infected T4 cells were all observed in LNs as drug penetration increased, though these values remain comparatively steady in PB. The influence of drug effectiveness on different types of cells is discussed in Table 4. The simulation results show that as the differences in inter-cell drug effectiveness decrease, decreased viral peak values and viral load were observed in both PB and LNs on day 6000. Both the total T4 cell count and infected T4 cell count at day 6000 were decreased in PB, but only the infected T4 cell count decreased in LNs. A slight increase in total T4 cell count was observed in LNs.

**Table 3:** Simulation of different levels of drug effectiveness in different compartments.

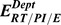	Double viral peaks	Viral load on day 6000	T4 cell count on day 6000	Infected T4 cell count on day 6000	Inf. T4/toatal T4
0	1116/5(2607/4)	250(115)	114(1839)	40(144)	35.1%(7.8%)
0.25	209/15(1292/*)	23(75)	523(5130)	150(2845)	28.7%(55.5%)
0.5	206/15(1026/*)	23(61)	520(5623)	149(2868)	28.7%(51.0%)
0.75	203/15(791/*)	23(49)	518(6320)	149(2926)	28.8%(46.3%)
1	200/15(595/*)	23(40)	516(7273)	149(3017)	28.9%(41.5%)

Note: The numbers in parentheses represent the results in LNs and those not in parentheses represent the results in PB.

* indicates the absence of second peak. Here 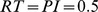, 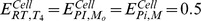, and BTT is day 0.

**Table 4:** Simulation of different levels of drug effectiveness in different cell types.

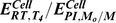	Double viral peaks	Viral load on day 6000	T4 cell count on day 6000	Infected T4 cell count on day 6000	Inf.T4/total T4
0	1116/5(2607/4)	250(115)	114(1839)	40(144)	35.1%(7.8%)
0.25	279/16(1200/*)	25(67)	554(5596)	177(3033)	31.9%(54.2%)
0.5	206/15(1026/*)	23(61)	520(5623)	149(2868)	28.7%(51.0%)
0.75	144/14(866/*)	22(55)	482(5641)	122(2683)	25.3%(47.6%)
1	93/*(725/*)	21(50)	440(5654)	95(2485)	21.6%(44.0%)

Note: The numbers in parentheses represent the results in LNs and those not in parentheses represent the results in PB.

* indicates the absence of a second peak. Here 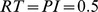, 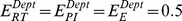, and BTT is day 0.

### Treatments initiated various days after infection, can not change patients' outcomes, accompanied by a relatively high Inf.T4/T4 ratio

Table 5 lists the dynamics of HIV-1 infection for BTT of day 1000, day 2000, day 3000, and day 4000, which correlate to the T4 cell counts of about 501 cells/mm^3^, 332 cells/mm^3^, 242 cells/mm^3^, and 182 cells/mm^3^ in PB, respectively. The simulation results demonstrate that suppression of viral replication with antiretroviral agents causes rapid recovery of the T4 cell count [63]. Early treatment can significantly increase the peak T4 cell count in both PB and LNs during infection (Table 5, second column). However, the total T4 cell count and infected T4 cell count on day 6000 remained relatively steady for different BTTs, especially in PB before the onset of AIDS (Table 5, third, fifth, and sixth columns). The viral loads on day 6000 were the same for all BTT scenarios (Table 5, fourth column).

**Table 5:** Simulation of different BTTs.

BTT	Peak T4 cell count	T4 cell count on day 6000	Viral load on day 6000	Infected T4 cell count on day 6000	Inf. T4/total T4
1000	688(8114)	520(5619)	23(61)	149(2864)	28.7%(51.0%)
2000	622(5680)	520(5604)	23(61)	149(2851)	28.7%(50.9%)
3000	559(5526)	518(5523)	23(61)	149(2779)	28.8%(50.3%)
4000	496(5126)	496(5126)	24(61)	147(2415)	29.6%(47.1%)

Note: The numbers in parentheses represent the results in LNs and those not in parentheses represent the results in PB. Here 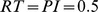, 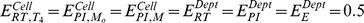.

Regarding BTT, it has been suggested that, relative to therapy that is delayed until the T4 cell count falls into the range of 200–250 cells/mm^3^, there is a clinical benefit to the initiation of antiretroviral therapy when a person has a T4 cell count of 350–550 cells/mm^3^ [64]. This can be seen from our simulation results of peak T4 cell count by comparing the first two rows of Table 5 to the third and fourth rows. There are still some discrepancies: First, in PB, the differences in T4 cell count, viral load, and infected T4 cell count after 6000 days of treatment are small under different BTT conditions. Second, although some significant improvements in the peak T4 cell count can be observed, the differences between the peak values and their initial cell count before treatment is small. Finally, there is a serious risk of reoccurrence of viral production because the ratio of infected T4 cells to total T4 cells has not changed significantly in either compartment.

Although with aforementioned differences, a recently published international cohort study shows that starting HIV therapy when a patient's immune system is still strong does not reduce the risk of AIDS or death [65]. This supports our simulation result that different BTT does not significant change patient's outcome.

## Discussion

In the present study, we proposed a novel dynamic model based on the biological mechanisms of immune response to HIV-1 infection. This model has three connected compartments, the PB, LNs, and CNS. The simulation results show that this model successfully simulates the three distinctive phases (acute infection, asymptomatic phase, and AIDS) of untreated HIV-1 infection. An additional drug response phase with HAART therapy is also applied. The simulation results also captured the typical features of the dynamics between the immune system and virus during HIV-1 infection, which has been observed in clinical patients in several ways: the double viral peaks during acute infection, the dramatic decline of total T4 cell count in the first viremia, the steady state between the immune system and virus, the inverted T8/T4 ratio, the hyper-activation of T8 cells, the three typical stages of the change in LNs during HIV-1 infection, and the role of LNs as a major location of viral replication and formation of latently infected T4 cells pool during latency period [66]. We particularly addressed the contribution and functional role of cell-cell spread of infection and the induction of T4 cell apoptosis in HIV-1 infection. Our simulation results match clinical observations in the following ways: Uninfected T4 cells are the major type of cells that die during the infection [51]. Apoptosis among uninfected T4 cells is caused indirectly but mainly by infected macrophages [51]. T4 cell infection spreads from cell interaction plays an important role in infection and this is mainly caused by FDC^i^ in LNs [17]. In addition to the well-documented results of HIV-1 infection, we also observed some new features, including triple T4 cell peaks in PB, the bottleneck value of T8/T4 ratio, and infected macrophages acting as major causes of T4 cell death and viral production.

Infected macrophages are the major cause of T4 cells death and viral production in our model. This contradicts Perelson's observations [67]. However, it is difficult to measure levels of macrophage-derived viruses within infected individuals in vitro, and opportunistic infections can increase the proportion of macrophage-derived virus to 10% or greater of plasma virus levels [68], [69]. This suggests that our results are reliable. In addition, it has been found recently that HIV-1 sensitizes the monocyte-derived macrophages to apoptosis in response to TNF-Related Apoptosis Inducing Ligand (TRAIL), which may compromise the role of macrophages in viral production [70].

It has been suggested that infected macrophages may be resistant to the cytopathic effects of the virus and to current antiretroviral therapies, allowing them to act as a viral reservoir [71], [72]. Therefore, we simulated the effects of infected monocytes/macrophages on the disease progression by means of setting the infection rate of monocytes/macrophages at zero. The simulation results show that the decline in T4 cell count during the first viremia is less pronounced in PB when infected monocytes/macrophages are removed from the system, but an increment of total T4 cell count in 6000 days was observed in both PB and LNs, about four times greater than in the natural course of infection in PB and three times greater in LNs. Within addition to the changes in T4 cell count, the viral load remained relatively low when infected monocytes/macrophages, were absent from both in PB and LNs. Combining the simulation results of macrophages' role in inducing T4 cell infection and death, we conclude that infected monocytes/macrophages play an important role in the outcome of the HIV-1 infection. For this reason, we propose a new pathway to control HIV-1 infection: if we can block or minimize the influence of those cells, the patients' T4 cell counts remain relatively high and their viral loads can be controlled, which may extend the patients' life expectancy.

We then simulated the effects of antiretroviral drugs on HIV-1 infection through a typical HAART regimen, which consisted of RTs and PIs. When treatment was administered at the onset of infection, accompanied by the increase of the drug efficiency, the outcome of the disease improved, as measured by the total T4 cell count and viral load after 6000 days. Nevertheless, the infected T4 cell count showed a significant increase after therapy in both PB and LNs. The increase in the number of infected T4 cells is more striking in LNs than in PB, as indicated by the ratio of infected T4 cells to total T4 cells after 6000 days of therapy. We evaluated the influence of drug effectiveness on different types of cells and in different compartments. As the inter-cell differences in drug effectiveness diminished, both the total T4 cell count and infected T4 cell count decreased in PB, but only the infected T4 cell count decreased in the LNs. We have simulated our model dynamics under different BTT conditions, and although early treatment was found to significantly increase the peak T4 cell count in both the PB and LNs, the total T4 cell count and infected T4 cell count remained relatively constant after 6000 days under different BTT conditions. The simulation results before the onset of AIDS in PB after 6000 days under different BTT conditions showed no differences with respect to viral load.

We can conclude that, under current HAART therapy, only very efficient drugs can significantly change patient outcomes, which here means extending their lives. Different BTT cannot do this, although some benefits could be seen if treatment begins before the onset of AIDS. In this way, the present results offer a theoretical proof of the recent international cohort study [65]. This proof can be described as follows. The ability of T4 cells to proliferate was strengthened for a short period of time after antiviral drug therapy. This was accompanied by attenuated viral infection of T4 cells and macrophages and an increased T4 cell count. Meanwhile, however, the pool of T4 cells capable of being infected is also increased, and the virus is readily able to infect T4 cells due to the evolution as the disease progression. Additionally, the compromised functional abnormalities of T4 cells, T8 cells, and macrophages cannot be restored through HAART, which means that the T8 cells of AIDS patients have a significantly reduced ability to kill infected cells. As a result, the T4 cell count increases notably during antiviral therapy, but most of the new cells are latently infected T4 cells. As the disease progresses, the function of various immune cells become increasingly less effective. Certain stimuli, such as opportunistic infections, can stimulate latently infected cells to produce virus. At this point, the immune system cannot control the virus, leading to the failure of therapy.

Additionally, based on several research observations, we may raise some new views in monitoring and therapy of HIV-1 infection, which may need further study and experimental testing.

1) Monocytes/macrophages dynamics are closely correlated to viral load and the progression of disease. This is especially true of their role in inducing T4 cell infection and apoptosis. Monocytes/macrophages may therefore suitable as a new monitor marker of HIV-1 infection.

2) The pool of latently infected T4 cells is considered one of the major impediments to HIV-1 eradication. When those cells become reactivated, the viral particles that are released can spread and infect healthy T4 cells. This reactivation process can in turn facilitate the continual replenishment of the T4 cell reservoir, offsetting the benefits of antiviral therapy and contributing to the persistence of HIV-1 [73]. To our knowledge, HIV-1-infected patients usually die along of opportunistic infection and reoccurrence of HIV-1 production. Infected T4 cells serve as a reservoir of HIV-1 virions, and increased number of these cells may be the cause of HAART failure. In this way, the detection of latently infected T4 cells might also be suitable as a new monitor marker for use in patient evaluation. There are already some methods that can achieve this [74].

3) The reduction of the differences in the levels of drug effectiveness between T4 cells and macrophages can significantly increase the total T4 cell count and limit the infected T4 cell count and the viral load in LNs. Because LNs are the major location of T4 cell depletion and viral replication, future drug development might focus on increasing drug efficiency and limiting the differences between these two types of cells.

Further studies are needed to explain the biological mechanisms underlying the results of the simulation, such as why different BTTs cannot induce substantive changes in the outcomes of the infected patients as suggested in a recent international cohort study [65]. The results shown here indicate that this model has the potential to provide insight into HIV-1 infection and drug treatment dynamics. However, because of the complexity of the interactions between HIV-1 and the immune system, refinements and improvements should be made to the model to incorporate more biological features of the disease into it. For example, some authors have postulated that the circulation of T4 cells between PB and LNs may be a cause of the T4 cell apoptosis and changes in T4 cell count in PB [75], [76]. In order to better understand HIV-1 infection, more detailed biological mechanisms should be contained in further mathematical models. Nevertheless, our new dynamic model can effectively capture the observed global features of HIV-1 infection, and has value in the analysis of the clinical progression and prognosis of HIV-1 infection.

## Methods

The present model is a modified version of a previously reported model [26]. These modifications are mainly related to natural biological immune processes, including the interactions between infected macrophages and T4 cells, the activation of T8 cells and macrophages, and the impact of viral load on the T4 cell proliferation. In our model, three compartments are named, the peripheral blood (PB), lymph nodes (LNs), and central nervous system (CNS). In order to produce a more realistic simulation of HIV-1 immune response, five types of cells were taken into account: T4 cells, T8 cells, monocytes/macrophages, B cells, and follicular dendritic cells. Cells are further grouped by function and state of infection.

In the first part, we develop a basic model of HIV-1 infection, including 35 equations for the simulation of the interaction between the virus and the host immune system. In the parameter description section, twelve parameters that vary over time are used with a rational description of their biological meanings. Then, we discuss the parameters values selection in parameter estimation section. These parameters are estimated using data from clinical experiments and previous research. Finally, based on the effects of antiretroviral HAART drugs, we discuss the improvement of our model using the administration of antiviral drugs and discuss current HAART therapy.

### Basic model during the course of a natural HIV-1 infection

**Figure 10:**
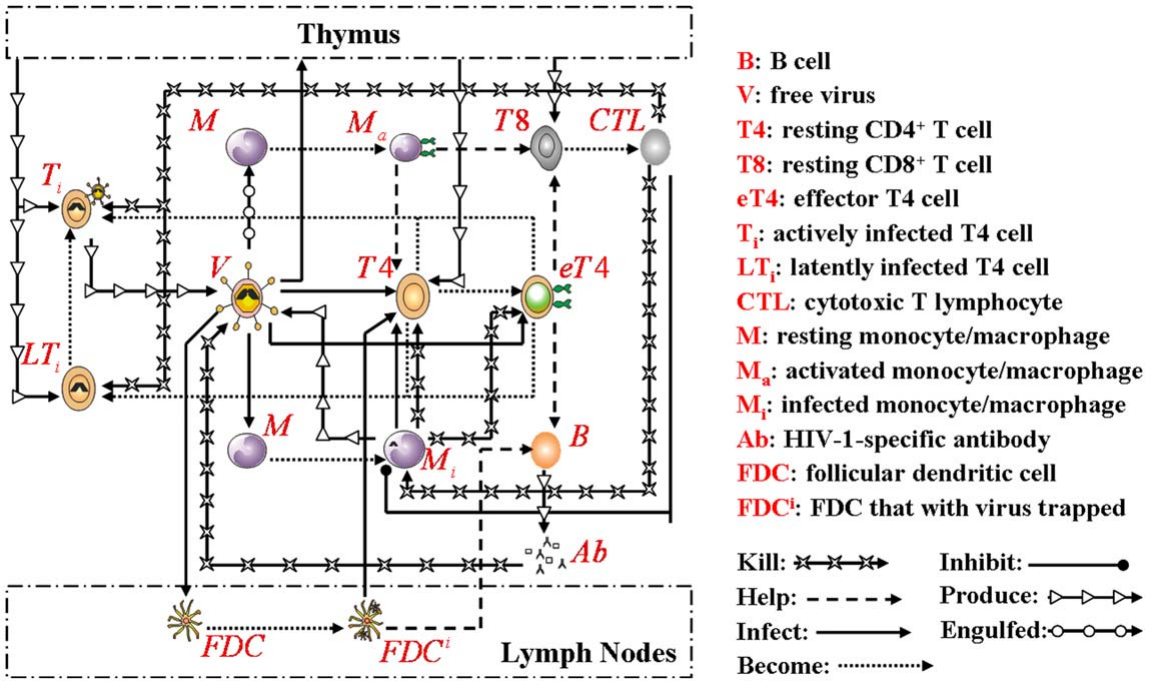
Immune response during HIV-1 infection. Viral particles could either be engulfed by monocytes/macrophages or infect those cells and T4 cells, causing a dramatic decline in T4 cells counts. After the immune recognition of HIV-1 antigens by activated monocytes/macrophages and the stimulation of HIV-1-specific T4 cell proliferation and differentiation, both the humoral and cell-mediated immunity responses took place. These are characterized by the proliferation of T8 cells and production of HIV-1-specific antibodies from B cells. T8 cells can kill infected T4 cells and monocytes/macrophages, and HIV-1-specific antibodies can inactivate free viruses and remove them. Free viral particles could either be produced from infected monocytes/macrophages or infected T4 cells. Viruses could also seed the thymus and LNs, causing the infection of newly produced T4 cells and forming a large virus pool in the LNs. In LNs, viruses are mainly trapped on the dendrites of FDCs.

Based on the biological mechanisms of immune response to HIV-1 infection in the host, the cell-virus and cell-cell interactions during HIV-1 infection can be summarized using the following road map of the immune response (Figure 10).

In the simulations, as in the previous study reference [26], the host was 36 years old with 1300 T4 cells/mm^3^ and 617 T8 cells/mm^3^ in PB when infected with 3 virions/mm^3^. The concentration of monocytes in PB was assumed to be 360 cells/mm^3^, as in the previous study [26]. The macrophages in PB were estimated to make up about 10% of total monocytes, which was the value used by Kirschner and Perelson [77].

Next, we calculated the initial T4 and T8 cells counts in LNs. The lymphocytes in the PB reflected only 2% of the total body T4 cells, and the rest mainly resided in the lymphoid tissues, which include LNs, adenoids, spleen, and the lymph [35]. Note that the volume of PB is about 5 L, and there are twice as many lymph vessels as blood vessels, so the volume of lymph in the body is about 10 L, twice that of blood [78]. Because there are about 500 LNs in the human body, and the size of each lymph node can be estimated at 1 cm^3^ [79], so the total volume of LNs per body is about 0.5 L. The volume of the adult spleen has been estimated to be about 0.2 L [80], [81]. In summary, the total volume of lymphoid tissues is about 11 L when the adenoids are taken into account. We assume that T4 cells are evenly distributed within the body and that the total T4 cell count is about 2×10^11^ cells [82]. This makes the concentration of T4 cells concentration in LNs about 1.8×10^4^ cells/mm^3^. In proportion, to be 2% of the total T4 cells, the T4 cell count in PB is about 4×10^9^ cells, and the cell concentration is 800 cells/mm^3^. Therefore, the concentration of T4 cells in the LNs is about 20 times than that in PB. Because the concentration of T4 cells in our model of PB is assumed to be 1300 cells/mm^3^, the cell concentration in LNs must be about 2.6×10^4^ cells/mm^3^. In the same way, the concentration of T8 cells has the same ratio with respect to PB and LNs. The concentration of T8 cells in the LNs is therefore about 12,340 cells/mm^3^, about 20 times that in PB.

In order to produce a detailed description of the dynamics of immune cells during the progression of the disease, T cells were classified as T4 cells and T8 cells, HIV-1-specific and -nonspecific cells, effector and noneffector cells. T4 cells were subdivided into healthy, latently infected, and actively infected cells. The concentrations of uninfected HIV-1-specific T4 and T8 cells are denoted by 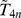, 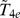, 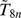, and 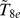, respectively. 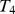 and 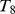 denote the concentrations of nonspecific cells. The subscripts 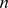 and 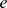 denote noneffector and effector cells, respectively. The superscript 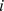 denotes latently infected cells, and the concentrations of latently infected HIV-1-specific T4 cells are denoted by 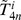 and 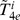. 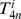 and 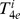 denote the concentrations of nonspecific latently infected T4 cells. The subscript 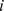 denotes actively infected T4 cells with the concentration 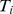. In order to differentiate the T4 cells and T8 cells in PB and LNs, we added the subscripts “PB” and “LN” as indicators of T4 and T8 cells. For example, 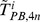 represents the concentration of latently infected HIV-1-specific noneffector T4 cells in PB, and 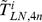 represents the concentration of the same kind of cells in LNs. Monocytes/macrophages are classified as resting, activated, and latently infected cells, and their concentrations are represented by 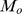 and 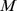, 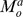 and 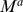, and 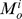 and 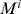, respectively. 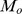 and 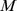 are assumed to be constant. 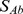 and 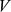 denote the concentrations of HIV-1-specific antibody and free virus, respectively. 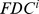 denotes the concentration of FDCs with virus and/or ICs trapped.

Our model incorporates two more compartments than the previous one, LNs and CNS. In order to produce a detail description of the dynamics of HIV-1 infection in these three compartments, we added an additional nineteen variables not evaluated in the previous study [26].

**Figure 11:**
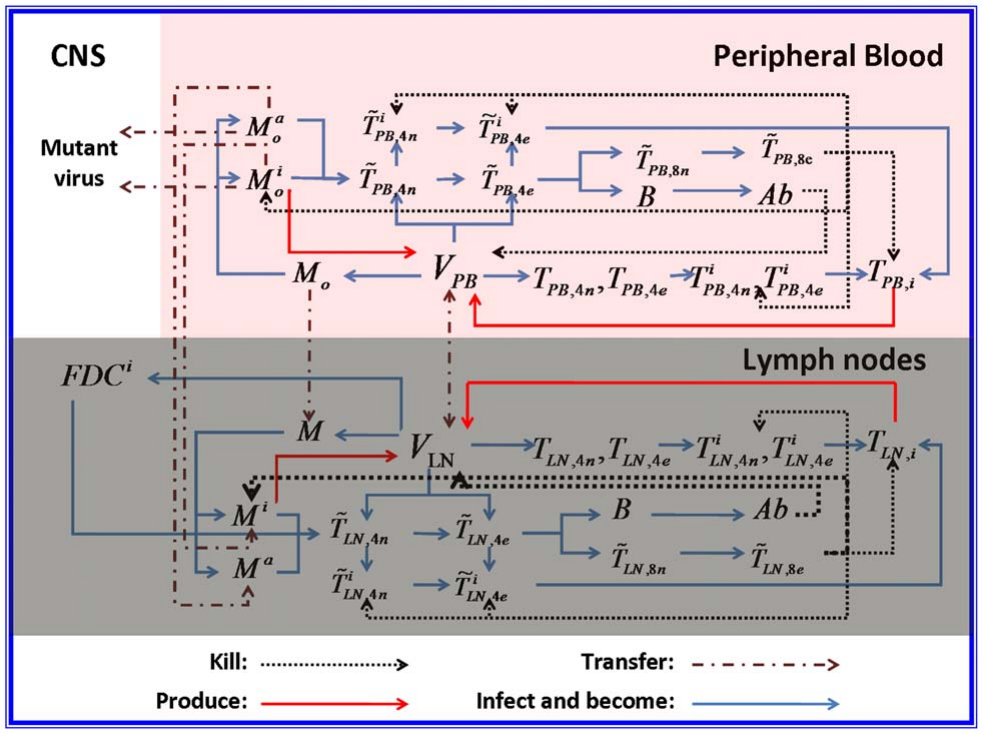
Global model dynamics. Three compartments, peripheral blood (PB), lymph nodes (LNs), and the central nervous system (CNS) are considered in this model. Interactions between different compartments took place mainly between PB and LNs and took the form of the migration of healthy, latently infected, and activated monocytes/macrophages and viral particles. Note that the migration of viral particles is bidirectional, but that of monocytes/macrophages is unidirectional. CNS is only considered with respect to the emergence and replication of mutant HIV-1 that could supplement the PB virus. All the variables shown in the Figure are detailed in Table 6.

Concretely, a global explanation of model dynamics including cell interactions, processes, and transfer between the main compartments is represented in the flow chart (Figure 11). Since the classification of T4 and T8 is same as that in the previous study and the previous study focus primarily on these cells in PB, we take the same initial values of different cell types in PB as [26]. For the initial values of different cell types in LNs, we can obtain through the above-mentioned proportion relationship respectively. In summary, all variables used in the equations (1) to (35) are summarized in Table 6, shown with their initial values and units. All the parameters used in our model are summarized in Table 7.

**Table 6:** Variables of the model and their initial values.

Variable	Definitions	Initial values in PB/LNs	Units
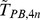 / 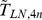	Healthy HIV-specific noneffector T4	0.92/18.4	cells/mm^3^
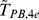 / 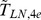	Healthy HIV-specific effector T4	0/0	cells/mm^3^
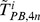 / 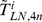	Latently infected HIV-specific noneffector T4	0/0	cells/mm^3^
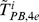 / 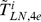	Latently infected HIV-specific effector T4	0/0	cells/mm^3^
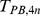 / 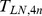	Healthy non-HIV specific noneffector T4	920/18400	cells/mm^3^
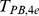 / 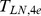	Healthy non-HIV specific effector T4	379/7580	cells/mm^3^
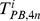 / 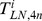	Latently infected non-HIV specific noneffector T4	0/0	cells/mm^3^
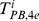 / 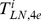	Latently infected non-HIV specific effector T4	0/0	cells/mm^3^
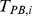 / 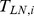	Actively infected T4	0/0	cells/mm^3^
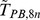 / 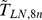	Healthy HIV-specific noneffector T8	0.47/9.4	cells/mm^3^
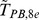 / 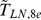	Healthy HIV-specific effector T8	0/0	cells/mm^3^
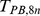 / 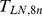	Healthy non-HIV specific noneffector T8	467/9340	cells/mm^3^
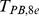 / 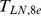	Healthy non-HIV specific effector T8	150/3000	cells/mm^3^
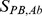 / 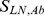	HIV-specific neutralizing antibody	0/0	molecules/mm^3^
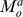	Activated HIV-specific monocyte	0	cells/mm^3^
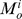	Latently infected HIV-specific monocyte	0	cells/mm^3^
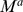	Activated HIV-specific macrophage	0	cells/mm^3^
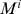	Latently infected HIV-specific macrophage	0	cells/mm^3^
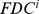	Follicular dentritic cell with HIV-1 trapped	0	cells/mm^3^
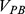 / 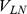	Virus	3/0	particles/mm^3^
	Viral RNA = virus×2000	3×2000 = 6000	molecules/ml

Notes: In this model monocytes only exist in PB and macrophages and FDCs only reside in LNs, so we did not differentiate those cells in the two compartments. Each HIV-1 virion has two RNA molecules in its nucleus, so the viral RNA molecules per milliliter is the number of viral particles per cubic millimeter times 2000.

**Table 7:** Parameters of the model and the estimated values.

Parameter	Definitions	Value	Reference
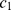	Michaelis-Menten half saturation for virus in PB	616.6 particles/mm^3^	[26]
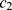	Michaelis-Menten half saturation for virus in thymus	1000 particles/mm^3^	[26]
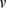	ST4/total T4 ratio in the thymic output	0.001	[92]
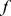	T4 percentage of neonate T cells	0.524	[26]
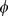	Fraction of T cells that differentiate	0.64	[26]
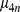	Death rate of 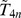 and 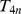	0.005/day	[26]
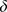	Recognition rate of latently infected T4, macrophages and monocytes by ST8	0.001	[26]
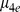	Death rate of 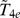 and 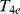	0.015/day	[26]
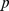	Fraction of latently infected T4 cells that become actively infected cells	0.03	[26]
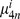	Death rate of 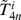 and 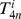	0.005/day	[26]
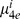	Death rate of 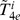 and 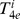	0.015/day	[26]
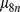	Death rate of 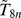 and 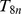	0.006/day	[26]
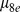	Death rate of 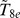 and 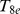	0.018/day	[26]
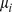	Death rate of actively infected T4 cells	0.47/day	[26]
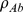	Antibody production rate per effector ST4	155 molecules/cell·day	[26]
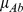	Death rate of antibodies	0.023/day	[26]
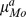	Death rate of 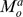	0.1/day	estimated
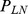	Fraction of monocytes migrating to LNs	0.4	estimated
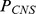	Fraction of monocytes migrating to the CNS	0.1	estimated
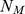	Viral production rate by 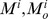	34 particles/cell·day	[93]
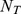	Number of virions produced by 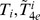 and 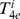 death	850 particles/cell	[26]
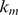	Rate of viral removal by macrophages/monocytes	60 particles/day	[26]
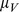	Death rate of free virus	3/day	[94]
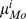	Death rate of 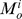	0.087/day	[26]
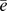	Rate of infection of thymic T4 cells at the time of infection	0.064/mm^3^·virion·day	[26]
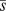	Flow of T cells from the thymus at the time of infection	6.09 cells/mm^3^·day	[26]
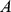	Age at inoculation	36 years	[95]
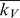	Rate of infection of T4 cells at the time of infection	0.089/mm^3^·virion·day	[26]
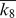	Rate of 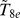 removes infected cells at the time of infection	2.5 mm^3^/cell·day	[26]
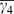	Proliferation rate of non-HIV specific T4 at the time of infection	0.0097/day	[26]
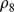	Proliferation rate of 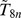 in presence of infected cells	0.36 mm^3^/cell·day	[26]
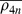	Proliferation rate of HIV-specific T4 in the presence of virus at the time of infection	1.98/day	[26]
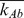	Rate of viral neutralization by HIV-specific antibody at the time of infection	0.01	estimated
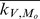	Rate of infection of healthy monocytes at the time of infection	1.19/day	[26]
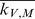	Rate of infection of healthy macrophages at the time of infection	1.19/day	[26]
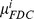	Death rate of FDC with virus bound	0.02/day	[96], [97]
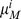	Death rate of 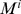	0.087/day	[26]
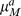	Death rate of 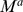	0.1	estimated
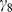	Proliferation rate of 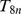	0.0091/day	[26]
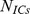	Initial number of ICs on FDCs	500	[98]
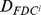	Time course of follicular network destruction	7000 days	estimated
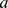	Coefficient of influence of V on uninfected T4 cell proliferation	1.05	estimated
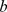	Coefficient of influence of V on latently infected T4 cell proliferation	3	estimated
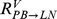	Fraction of virus outflow from PB into LNs	0.3	estimated
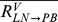	Fraction of virus outflow from LNs into PB at the time of infection	0.1	estimated
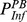	Probability of infected macrophages infect T4 cells in PB	4%	estimated
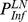	Probability of infected macrophages infect T4 cells in LNs	5%	estimated
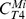	Coefficient of infected macrophages and T4 cells contact	0.004	estimated
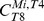	Coefficient of infected macrophages, effector T4 cells and T8 cells contact	0.0027	estimated

### Model of peripheral blood

Our PB dynamic model is similar to that used in a previous report [26]. However, we included an additional cell type (activated monocytes/macrophages) and some biological interactions such as cell-cell spread of infection and the induction of apoptosis. Here we mainly list the differences between our model and the previous model [26].

Eqs. (1)–(8) represented the dynamics of T4 cells, including specific and nonspecific, infected and uninfected, effector and noneffector T4 cells. The biological phenomena and flows considered in these equations are the effect of virus on the T4 cell proliferation, the induction of T4 cell infection by infected macrophages, the induction of infected T4 cell death by effector T8 cells and infected macrophages. The main differences between our model and the previous model are discussed.

The second term in Eq. (1) describes the proliferation of HIV-1-specific T4 cells in the presence of free virus. The previous study treated the proliferation rate 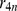 as a constant [26]. In our model, it is an assigned function of time. It has been observed that the maximal proliferation rate of T4 cells decreases as the disease progresses [83], [84]. Clinical observation shows that HIV-1-specific T4 cells exist in a partially anergized state under high viremia levels, that they lose their ability to proliferate in response to antigen exposure [49]. However, low viral load can stimulate immune proliferation and the influence of viral concentration on T4 cell proliferation is biphasic. Therefore, the effect of viral load on the HIV-1-specific T4 cell proliferation can be described by 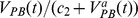 in Eq. (1), and 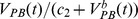 in Eq. (3), with the restriction that 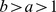.

The last two terms in Eq. (1) describe the effects of infected macrophages on HIV-1-specific T4 cells, including the induction of cell infection and apoptosis. Notably, the antigen-presenting function of monocytes is much lower than that of macrophages, which may impair its ability to induce T4 cell infection and apoptosis. Because the macrophages in PB are estimated to be about 10% of the total monocyte count, we add 0.1 to represent the number of macrophages that can functionally interact with T4 cells. We use 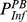 to describe the fraction that causes T4 cell infection, and it has a probability 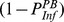 of inducing apoptosis. We also assume that the macrophage-induced T4-cell death rate is equal to the T4-cell natural death rate 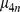. Parameter 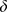 represents the rate of recognition of T4 cells by infected macrophages. These infected T4 cells are moved to Eq. (3) as a source of latently infected cells.

In Eqs. (3) and (4), HIV-1-specific CTLs effector cells eliminate infected cells by modulating the removal rate, and the coefficient 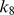 is weakened by 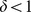 due to the lower rate of recognition of latently infected cells. It was assumed to be a constant in previous study [26]. In our model, the removal rate 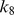 varies over time with the recognition of HIV-1-infected T4 cells by CTLs in an MHC-I restricted fashion, and MHC-I molecules are down-regulated during HIV-1 infection. This is mainly caused by HIV-1 protein negative regulatory factor (Nef) [47], [85], [86]. However, in Eqs. (4) and (8), the rate of killing of latently infected effector T4 cells by CTLs is not decreased, and those effector cells have more active functions and are recognized by CTLs relatively easily. Those cells are therefore considered actively infected cells and capable of producing virus. They are moved to Eq. (13), in contrast to a previous study [26].

The equations for non-specific T4 cells are similar to Eqs. (1)–(4), with a proliferation rate 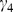, which takes into account the background average stimulation by their antigens. It varies over time because viremia influences the proliferation rate of T4 cells [83], [84]. In the previous study, it was assumed to be constant [26].
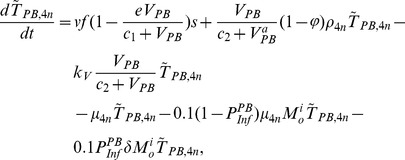
(1)

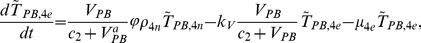
(2)

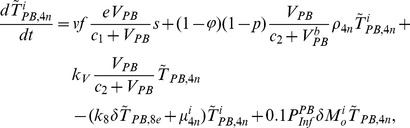
(3)

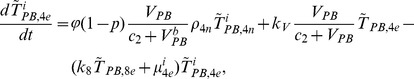
(4)

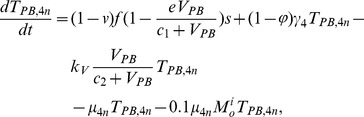
(5)

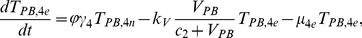
(6)

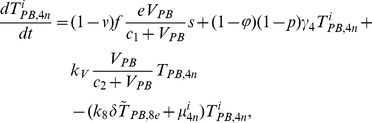
(7)

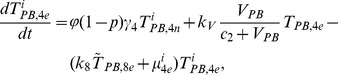
(8)
Eqs. (9)–(12) model the dynamics of HIV-1-specific and –nonspecific T8 cells. These are analogous to the equations of T4 cells. The main difference is that T8 cell proliferation is stimulated with the help of activated macrophages and HIV-1-specific T4 cells. The quantity 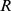 is the proliferation rate of specific T8 cells, which is defined in Eq. (46).
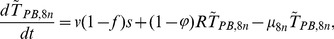
(9)

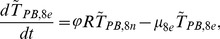
(10)

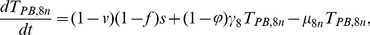
(11)

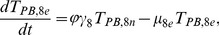
(12)
Eq. (13) shows the dynamics of actively infected T4 cells that can produce new viral particles. These particles are derived from the latently infected noneffector T4 cells. Eq. (14) describes the dynamics of B cells that produce HIV-1-specific antibody. The production of HIV-1-neutralizing antibodies is controlled by the HIV-1-specific effector T4 cells, both infected and uninfected.
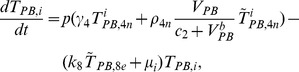
(13)

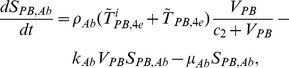
(14)
Eqs. (15) and (16) represent the dynamics of activated and infected monocytes. The activation of resting monocytes (whose concentration is 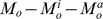), requires the phagocytosis of pathogens and is controlled by viral load, which is described as 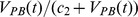 in Eq. (15). The second term represents the natural death of activated monocytes. The third term is the description of monocyte migration to other tissues, though the CNS and LNs are the only two considered here. In Eq. (16), the infection of healthy monocytes (whose concentration is 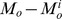) is described at rate 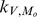, which is time-dependent. The second term represents natural death. Infected monocytes can be recognized and removed by CTLs. this process is described in the third term. The migration of infected monocytes is considered in the fourth term, which is similar to Eq. (15).
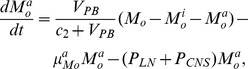
(15)

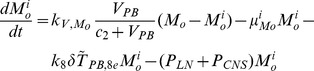
(16)The last equation, Eq. (17), describes the dynamics of free virus. Newly produced viral particles come either from infected macrophages or infected T4 cells. These are shown in the first two terms. Note that the latently infected effector T4 cells are also taken into account, as discussed in Eqs. (3)–(4). The last two terms represent the communication of virus between PB and LNs. Viral outflow from PB is assumed as a constant rate 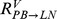, and the rate from LNs into PB is 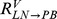. This increases over time and represents the increased outflow of virus to PB along with the destruction of LN architecture.
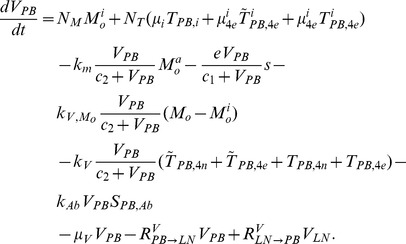
(17)


### Model of lymph nodes

The basic immune response in LNs is similar to that in PB, but it involves an additional cell type (FDCs). Eqs. (18)–(35) describe the model dynamics in LNs, and Eq. (35) represents the dynamics of FDCs. The first term represents healthy FDCs (those without HIV-1 or any reduction in antigen-binding ability, described as 
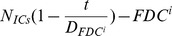
). These cells trap virus at a constant rate 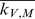, which is controlled by viral load. Here 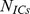 represents the initial number of ICs deposited on FDCs before infection, and 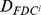 represents the time course of follicular network destruction due to HIV-1 infection. The second term describes the natural loss of ICs from FDCs. In addition, we have assumed that each FDC has about twenty dendrites and that it takes 20 viruses to infect one FDC [87]. The deposition of virus in the FDCs network could be considered one way of removing free virus. This is reflected in Eq. (34) in the sixth term.
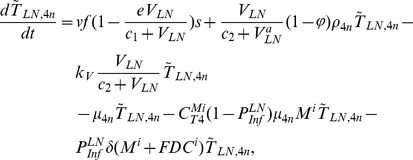
(18)

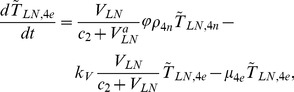
(19)

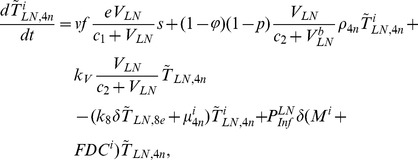
(20)

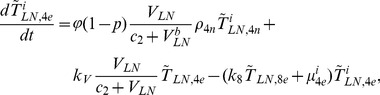
(21)

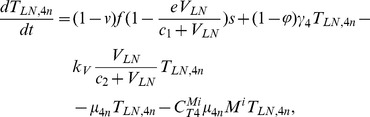
(22)

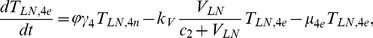
(23)

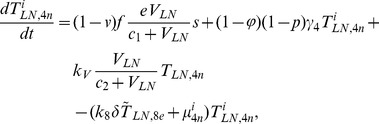
(24)

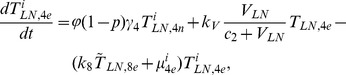
(25)

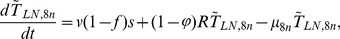
(26)

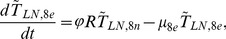
(27)

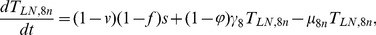
(28)

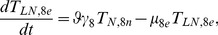
(29)

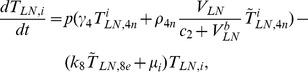
(30)

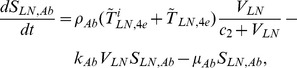
(31)

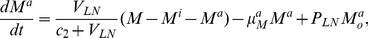
(32)

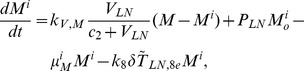
(33)

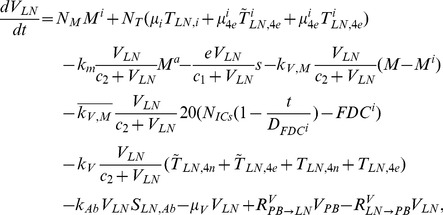
(34)

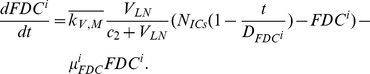
(35)


### Model of the central nervous system

The progression of this disease within CNS is largely dependent on the continuous seeding of the activated leukocytes from PB. These are mainly monocytes/macrophages. We describe this effect in Eqs. (15) and (16), which represent the migration of activated and infected monocytes/macrophages from PB to the CNS and LNs. Note that CNS acts as a sanctuary compartment during HIV-1 infection. There, viral mutants emerge. Because we have taken into account the evolution and mutation of virus and their effects during the infection in our model of PB and LNs, we did not list the formula of CNS in our dynamic model.

### Description of parameters

The parameters 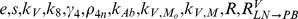 vary over time according to their assigned functions, and 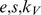 have been described previously [26].
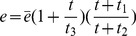
(36)

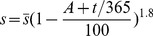
(37)

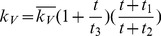
(38)

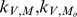
, and 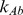 decrease over time. They represent the increasing tropism of viruses for T4 cells and the decreasing removal rate of ICs. This decrease takes place because mutated viruses can escape antibody neutralizing function [88], which are shown in Eqs. (39)–(41). However, they are all believed to increase over time [26]. We think these are inappropriate for use as references for the clinical observation.
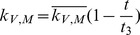
(39)

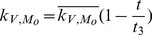
(40)

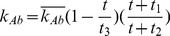
(41)

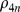
 and 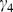 represent the influence of viral load on the basic proliferation rate of HIV-1-specific and -nonspecific T4 cells. Those two parameters are assumed to remain constant [26]. We prefer them for the time varying mode, as in studies that have shown that the ability of T4 cells to proliferate is absent or reduced after acute infection [29], [49], [50].
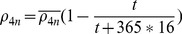
(42)

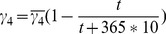
(43)


The rate of removal of infected cells by HIV-1-specific effector T8 cells is given in Eq. (44). This rate is assumed to remain constant [26]. However, the function of T8 cells is impaired during infection [44],[45,[46],[47],[48]. This is due to the absence of HIV-1-specific T4 cell proliferation and function, which are needed for the activation of T8 cells. This reduces the ability of T8 cells to kill infected cells. A time varying decreasing function of 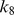 was therefore assumed.
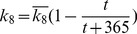
(44)Continuous deconstruction of LNs structure can be observed during HIV-1 infection [33], [34]. This disruption may facilitate the outflow of newly produced viruses from LNs into the PB. An increasing time function can be used to describe the migration rate of virus from LNs into PB, 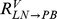, with an initial value of 0.1.
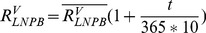
(45)Here the expression 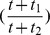 represents the increasing T-tropism of virus for T4 cells. As described previously [26], the values of 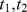, and 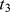 are estimated as 1 day, 1000 days, and 40,000 days. These are the times required to reproduce a reasonable delay in the adaptation of tropism and a very mild increase in the basic infectiveness. These all remained constant throughout the simulation.


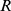
 is the proliferation rate of HIV-1-specific T8 cells in the presence of infected cells. T4 cell function is required for sustained CTL response to HIV-1, which can help T8 cells through cell-cell interactions and increase expression of costimulatory molecules, such as B7-1 and B7-2, on the antigen-presenting cells [89]. Based on the biological processes described above, the proliferation rate 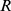 of T8 cells in Eqs. (9) and (10) can be set as follows:
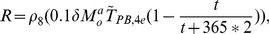
(46)and in Eqs. (26) and (27) are given as follows
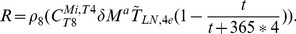
(47)Here 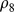 is proportionality constant of T8 proliferation rate. The coefficient 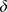 expresses the reduced capability of recognition of latently infected T4 cells and infected macrophages during the infection. The ability of infected T4 cells and macrophages to stimulate other cells declines gradually during the course of infection due to their functional abnormalities. Therefore, the coefficient 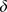 is a time-dependent, decreasing function, and we describe this phenomenon by multiplying 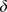 by 
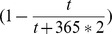
 and 
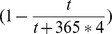
 for PB and LNs, respectively. The fact that there are more T4 cells and macrophages in LNs than in PB may compensate for the reduction in the rate of stimulation of proliferation of T8 cells, we assumed that LNs decrease at a slower rate.

### Parameter estimation

The values of 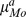 and 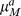, the death rates of activated monocytes and macrophages, were estimated at 0.1/day. Because the life-span of activated macrophages ranges from 6 to 16 days [90], we used an estimate of 10 days. Therefore, the death rate is 0.1/day. For the death rate of infected monocytes, 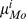, we chose the value used for infected macrophages, 0.087/day.

Probabilities of infected macrophages infect T4 cells in PB and LNs, 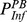 and 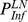, were set at 4% and 5% respectively. Because the T4-cell functional abnormality occurs early during infection, with the increasing HIV-1 proteins inducing apoptosis in conjunction with the role of infected macrophages in T4 cell death, the interaction between T4 cells and infected macrophages may preferentially cause T4 cell anergy and apoptosis, so we assume 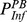 equals 4% and 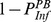 to be induced apoptosis in PB. It has been suggested that the virus preferentially causes latently infected T4 cells pool in LNs during infection. The value 5% was chosen for 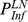.

For the coefficient of infected macrophages and T4 cells that make contact in the LNs, 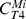, an assumed value 0.004 was chosen. In LNs, the distribution of macrophages and T4 cells is different, and cell movement meets more resistance than in PB. In addition, after monocytes migrate into LNs, it takes a moment for them to differentiate into macrophages and develop the antigen-presenting function. Considering this, we estimated the value at about 0.004. The estimation of coefficient of infected macrophages, effector T4 cells, and T8 cells interaction, 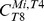, was performed in the same way as that of 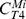, although with a slight decrease, 0.0027, because it requires contact between three cells.

The fraction of viruses that migrate from PB into LNs, 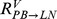, is estimated to be 0.3. It has been hypothesized that the virus enters the CNS mainly through infected monocytes and macrophages, so the migration of free virus is only considered in the case of LNs.

The rate of monocyte migration from PB into LNs and CNS is estimated to be 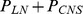, with a total value 0.5 [91]. Because the LNs are the major sites of antiviral response, and take the lower infiltration of blood-brain barrier into account, 0.4 and 0.1 were chosen for 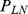 and 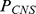, respectively.

Through the natural course of HIV-1 infection, FDCs trap and retain large numbers of viruses. As the disease progresses, destruction of FDCs and the follicular architecture can be seen in secondary lymphoid tissues. In order to describe the role of FDCs in cell-spread infection and follicular destruction during HIV-1 infection, we assumed a parameter 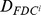 to represent the time course of the destruction of the FDC network. A value 7000 days was chosen according to our simulation scale of 6000 days.

The variables 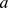 and 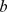 are the influence of viral load on the proliferation of uninfected and latently infected T4 cells, respectively. These were set at 1.05 and 3, respectively. With higher viral load, viruses preferentially inhibit uninfected T4 cell proliferation. When viral load decreases, viruses inhibit latently infected T4 cell proliferation in an effort to sustain the pool of latently infected T4 cells [66]. In order to describe such effects, a larger value of 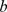 is assumed.

Other parameters are taken from relevant references because all the common parameters play exactly the same role in both models. The parameters, their initial values, units, and references are listed in Table 7.

### HAART-treated model

With the description of HIV-1 infection provided in the basic model and with the aim of a better understanding of the mechanisms underlying HIV-1 infection, we simulated a model of reactions to antiviral drug therapy. There is no cure for AIDS, but drugs can be used to control the virus and its complications. Antiretroviral drugs target multiple steps in the viral life cycle, including fusion, reverse transcription, and the protease processing required for viral infectivity. Anti-HIV drugs are classified by mechanism: non-nucleoside reverse transcriptase inhibitors (NNRTIs), nucleoside reverse transcriptase inhibitors (NRTIs), protease inhibitors (PIs), entry and fusion inhibitors, and integrase inhibitors [99]. A combination of those antiretroviral drugs, called highly active antiretroviral therapy (HAART), has been very effective in reducing the number of HIV-1 particles in the blood [100]. Typical HAART regimens consist of two NRTIs plus either a PI or a NNRTI. HAART is thought to increase survival time by between 4 and 12 years [101]. RTIs block the translation of viral RNA into DNA for incorporation into the host genome, preventing the infection of new cells. In contrast, PIs interfere with essential steps of protein cleavage in new virions, preventing infected cells from producing infectious viral particles [102].

Although they involve the same inhibitor mechanisms, lymphocytes and monocytes/macrophages provided distinct milieus for the effectiveness of antiretroviral inhibitors. RTIs are more effective in macrophages than in T4 cells. In contrast, PI suppression of viral replication in macrophages requires significantly higher levels of inhibitor than levels known to be effective in lymphocytes [103]. It has recently been suggested that many of the mutations commonly occurred during therapy do not have a direct connection with drug resistance [104]. This convinced us to simulate a drug administration model without changing the viral mutation rate.

According to the effects of the drugs described above, the associated parameters have been changed in the following respects.

In order to describe the reduction in the rate of infection of new cells caused by RTIs, the rates of infection of T4 cells and monocytes/macrophages, 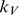 and 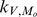/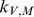, are multiplied by 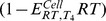 and 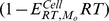 in PB and by 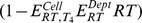 and 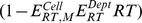 in LNs. Here 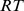 is a constant that represents the effectiveness of RTI inhibition, the efficiency of the drug. 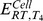 and 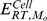/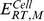
describe the different levels of effectiveness of RTIs in T4 cells and monocytes/macrophages. Because RTIs are highly effective in macrophages, we set 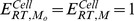 for simplicity and let 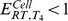 represent the lesser effectiveness of RTIs on T4 cells. 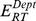 represents the different concentrations of drugs in the two compartments, PB and LNs. According to the assumption that the drug is initially saturated in PB and then penetrates into the LNs, here we only add 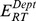 to the LNs equations and let 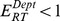.

PIs prevent infected cells from producing infectious viral particles, and we describe this effect by changing the production rate of virus in Eqs. (17) and (34). This effect is similar to the RTIs, with the exception that PIs are more effective in T4 cells. Let 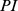 represents the effectiveness of the inhibition of PIs. The viral production rates of infected T4 cells and infected monocytes/macrophages are multiplied by 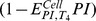 and 
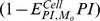
 in PB, and by 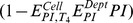 and 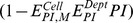 in LNs, respectively. This time, we set 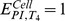, and let 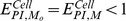 because PIs are more effective in T4 cells. PI affects immune reconstitution in a manner independent of its ability to suppress HIV-1 replication, as indicated by the fact that the decrease in apoptosis occurs before any significant changes in plasma levels of viral RNA when PI is used alone [105], [106]. In order to describe the effect of PIs on T4 cell count, the rate of T4 cell apoptosis caused by infected macrophages, 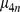, is multiplied by 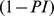 in PB and 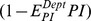 in LNs. Again, we assume that 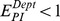 represents the difference in drug concentrations between the two compartments.

During HIV-1 infection, the proliferation rate of HIV-1 nonspecific T4 cells decreases over time, but with the administration of drugs and the reconstitution of the immune system, this value is somewhat restored. We simply represent this effect by changing the expression of 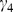 to the equation 
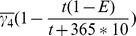
 in PB and 
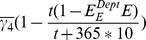
 in LNs, with a time delay 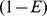 and 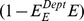 added in each part. This describes the slow decrease of the proliferation rate when drugs are used. The coefficient 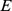 is the mean value of 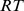 and 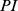, 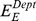 describes the difference in drug concentration between those two compartments, as above, and 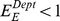.

The formulas used in our HAART-treated model are provided in File S1.

## Supporting Information

Figure S1 Dynamics of infected cells in PB and LNs, including latently and actively infected T4 cells and infected monocytes/macrophages. Panel A represents the situation in PB, and panel B represents it in LNs. Both panels show that the infected cells reach their maximal values in conjunction with the viral peaks. However, there are some componential differences during the first viral peak. During the first viremia, monocytes are primary type of infected cell in PB, and latently infected T4 cells are the major type of infected in LNs. After that, monocytes/macrophages are the major type of infected cell. The results of the simulations support the following conclusions: During the initial HIV-1 infection, most viruses are M-tropic, using CCR5 coreceptor for viral attachment. This coreceptor is extensively expressed on the surface of macrophages, allowing the virus infect macrophages more easily than T4 cells. This can be seen in PB in the results of our simulation. In the LNs, latently infected T4 cells are the major type of cell infected during the first viremia. After that, infected macrophages are more common. In both PB and LNs, we can see that actively infected T4 cells are rarer than latently infected T4 cells, which means that infection of T4 cells by HIV-1 preferentially induces latent infected T4 cells pool, especially in LNs [66]. The infection of T4 cells increases gradually throughout the asymptomatic and AIDS phases, which represents the increasing tropism of virus to infect T4 cells. The rate of infection of monocytes/macrophages also increased. This can be explained by the middle stage of evolution of viral tropism converting from R5-tropic to X4-tropic, the R5X4-tropic variants. This kind of variant can infect both T4 cells and monocytes/macrophages [54]. It has been suggested that although HIV-1 usage of CXCR4 develops over time in many individuals, R5-tropic strains predominate in chronically HIV-1-infected patients and cause T4 cell depletion [55]. This could also explain our simulation results.
(TIF)

Figure S2 Daily death of T4 cells in PB and LNs. There were two peak counts of cell death. These were accompanied with the double viremia in both PB and LNs, reaching values of about 32.7 cells/mm^3^ per day and 175.3 cells/mm^3^ per day at 9 days, and 0.037 cells/mm^3^ per day and 0.873 cells/mm^3^ per day at 113 days. After that, the numbers continued to increase gradually in both compartments, and a third peak count was observed in LNs, about 1.414 cells/mm^3^ per day at 435 days. During the AIDS phase (3668 days), the numbers reached 0.104 cells/mm^3^ per day and 1.07 cells/mm^3^ per day in PB and LNs, respectively.
(TIF)

Figure S3 Dynamics of activated and infected monocytes/macrophages in PB and LNs. Panel A represents the situation in PB, and panel B represents it in LNs. For the activated monocytes/macrophages, three cell count peaks were observed in both PB and LNs, and the cell counts continued to increase gradually during the chronic phase and into the AIDS phase. Activated monocytes/macrophages are required for CTL's cell-killing function, so it is reasonable that the proliferation of HIV-1-specific T8 cells may take place later than that of activated monocytes/macrophages. This is reflected in our results in the days on which those cells reached their maximum counts. For example, the first peaks of effector ST8 cell count in PB and LNs occur at 64 days and 79 days (Figure S3 **A** and S3**B**). For activated monocytes/macrophages, the first top values are both reached in 6 days. Because there are three peak counts of activated monocytes/macrophages, there are likely to be three peaks of ST8 cell count. However, only two peaks were observed in both PB and LNs in Figure S3. This might represent the inability of T4 cells to help CTL response formation, as suggested in previous studies [83]. A bottle-neck was observed among activated macrophages in LNs (Figure S3 **B**). According to the biological cell interactions mentioned above, the T8 cells should reach their top cell count after that, validating the hypothesis that the T8/T4 ratio should also reaches its peak during the disease progression in LNs. Unlike that of activated monocytes/macrophages, the dynamics of infected monocytes/macrophages showed only two peaks in PB andAll occurred in conjunction with double viral peaks.
(TIF)

File S1 Formula of HAART-treated model.
(DOC)
