# Supplementary figures and images for: Modeling of HIV-1 Infection: Insights to the Role of Monocytes/Macrophages, Latently Infected T4 Cells, and HAART Regimes
Source: PLoS One. 2012 Sep 26;7(9):e46026. doi: 10.1371/journal.pone.0046026 (PMC3458829; doi:10.1371/journal.pone.0046026)

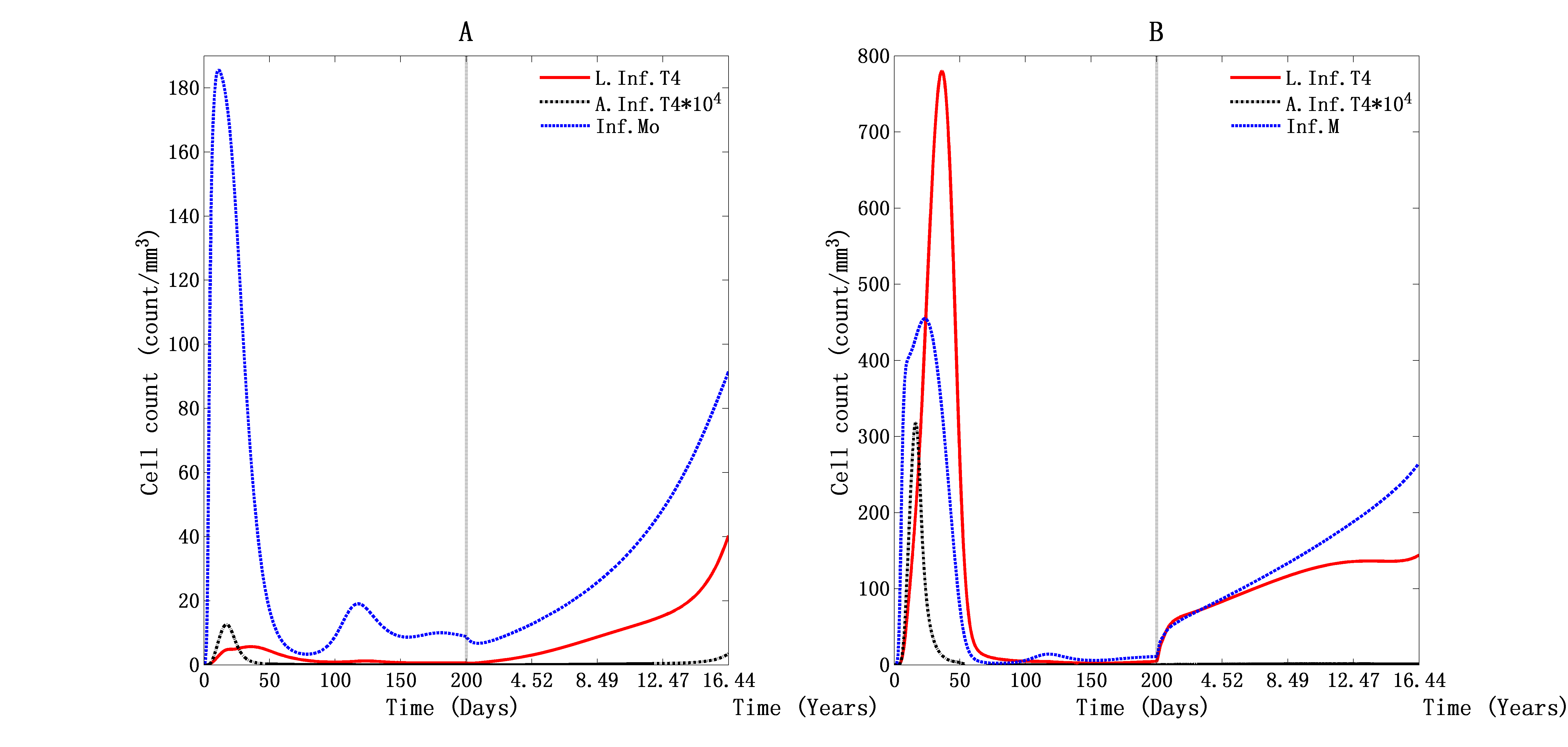

Supplement: Figure S1 — Dynamics of infected cells in PB and LNs, including latently and actively infected T4 cells and infected monocytes/macrophages. Panel A represents the situation in PB, and panel B represents it in LNs. Both panels show that the infected cells reach their maximal values in conjunction with the viral peaks. However, there are some componential differences during the first viral peak. During the first viremia, monocytes are primary type of infected cell in PB, and latently infected T4 cells are the major type of infected in LNs. After that, monocytes/macrophages are the major type of infected cell. The results of the simulations support the following conclusions: During the initial HIV-1 infection, most viruses are M-tropic, using CCR5 coreceptor for viral attachment. This coreceptor is extensively expressed on the surface of macrophages, allowing the virus infect macrophages more easily than T4 cells. This can be seen in PB in the results of our simulation. In the LNs, latently infected T4 cells are the major type of cell infected during the first viremia. After that, infected macrophages are more common. In both PB and LNs, we can see that actively infected T4 cells are rarer than latently infected T4 cells, which means that infection of T4 cells by HIV-1 preferentially induces latent infected T4 cells pool, especially in LNs [66]. The infection of T4 cells increases gradually throughout the asymptomatic and AIDS phases, which represents the increasing tropism of virus to infect T4 cells. The rate of infection of monocytes/macrophages also increased. This can be explained by the middle stage of evolution of viral tropism converting from R5-tropic to X4-tropic, the R5X4-tropic variants. This kind of variant can infect both T4 cells and monocytes/macrophages [54]. It has been suggested that although HIV-1 usage of CXCR4 develops over time in many individuals, R5-tropic strains predominate in chronically HIV-1-infected patients and cause T4 cell depletion [55]. This cou [file pone.0046026.s001.tif]

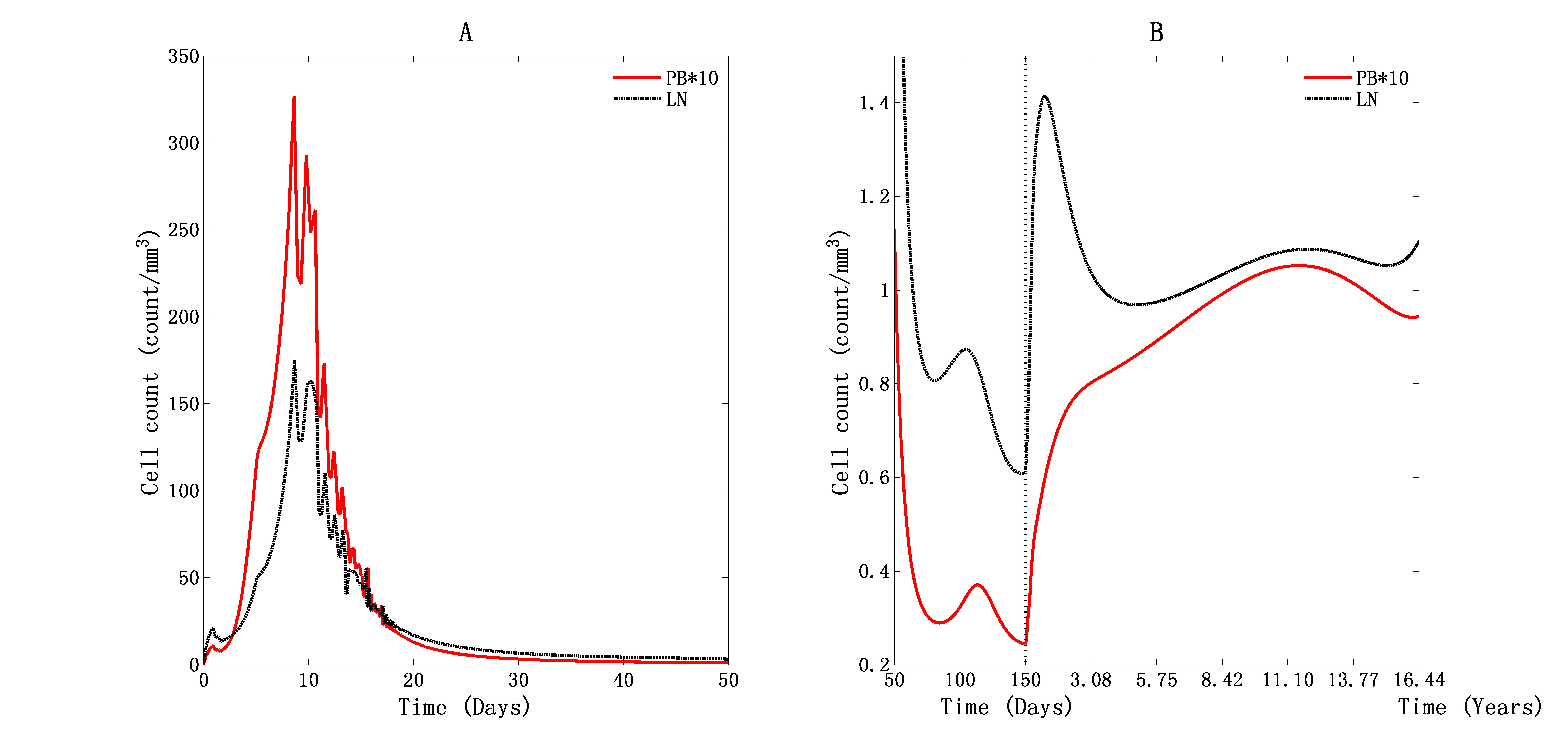

Supplement: Figure S2 — Daily death of T4 cells in PB and LNs. There were two peak counts of cell death. These were accompanied with the double viremia in both PB and LNs, reaching values of about 32.7 cells/mm3 per day and 175.3 cells/mm3 per day at 9 days, and 0.037 cells/mm3 per day and 0.873 cells/mm3 per day at 113 days. After that, the numbers continued to increase gradually in both compartments, and a third peak count was observed in LNs, about 1.414 cells/mm3 per day at 435 days. During the AIDS phase (3668 days), the numbers reached 0.104 cells/mm3 per day and 1.07 cells/mm3 per day in PB and LNs, respectively. (TIF) [file pone.0046026.s002.tif]

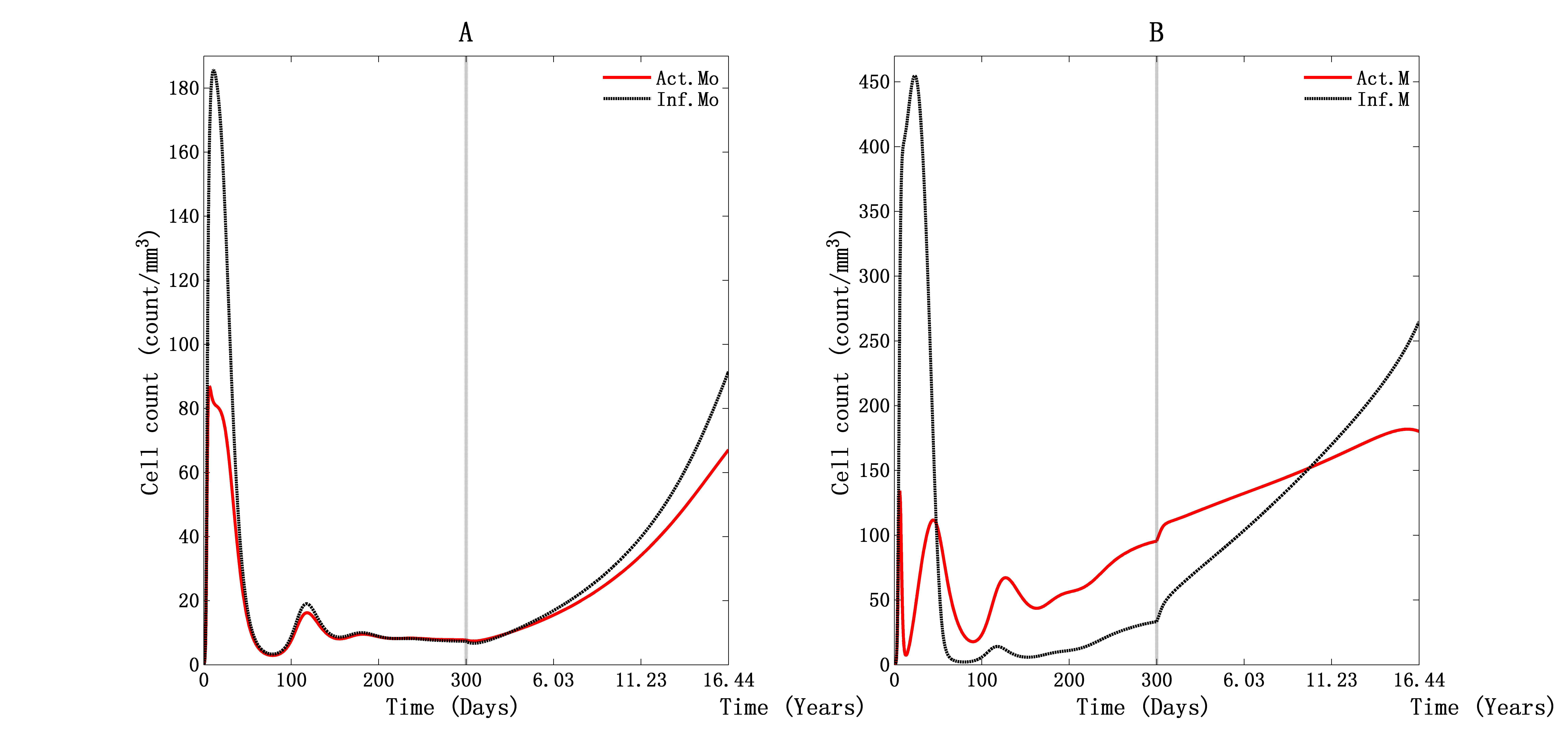

Supplement: Figure S3 — Dynamics of activated and infected monocytes/macrophages in PB and LNs. Panel A represents the situation in PB, and panel B represents it in LNs. For the activated monocytes/macrophages, three cell count peaks were observed in both PB and LNs, and the cell counts continued to increase gradually during the chronic phase and into the AIDS phase. Activated monocytes/macrophages are required for CTL's cell-killing function, so it is reasonable that the proliferation of HIV-1-specific T8 cells may take place later than that of activated monocytes/macrophages. This is reflected in our results in the days on which those cells reached their maximum counts. For example, the first peaks of effector ST8 cell count in PB and LNs occur at 64 days and 79 days (Figure S3 A and S3B). For activated monocytes/macrophages, the first top values are both reached in 6 days. Because there are three peak counts of activated monocytes/macrophages, there are likely to be three peaks of ST8 cell count. However, only two peaks were observed in both PB and LNs in Figure S3. This might represent the inability of T4 cells to help CTL response formation, as suggested in previous studies [83]. A bottle-neck was observed among activated macrophages in LNs (Figure S3 B). According to the biological cell interactions mentioned above, the T8 cells should reach their top cell count after that, validating the hypothesis that the T8/T4 ratio should also reaches its peak during the disease progression in LNs. Unlike that of activated monocytes/macrophages, the dynamics of infected monocytes/macrophages showed only two peaks in PB andAll occurred in conjunction with double viral peaks. (TIF) [file pone.0046026.s003.tif]
