# Supplementary material for: Modeling of HIV-1 Infection: Insights to the Role of Monocytes/Macrophages, Latently Infected T4 Cells, and HAART Regimes
Source: PLoS One. 2012 Sep 26;7(9):e46026. doi: 10.1371/journal.pone.0046026 (PMC3458829; doi:10.1371/journal.pone.0046026)
Supplement: File S1 — Formula of HAART-treated model. (DOC) [file pone.0046026.s004.doc]

Formula of HAART-treated model

(48)

(49)

(50)

(51)

$\begin{matrix} \frac{dT_{PB,4n}}{dt}=(1-v)f(1-\frac{eV_{PB}}{c_{1}+V_{PB}})s+(1-\phi)\gamma_{4}T_{PB,4n}-k_{V}(1-E_{RT,T_{4}}^{Cell}RT)\frac{V_{PB}}{c_{2}+V_{PB}}T_{PB,4n} \\ \begin{matrix} & & & -\mu_{4n} \end{matrix}T_{PB,4n}-0.1(1-PI)\mu_{4n}M_{o}^{i}T_{PB,4n}, \end{matrix}$ (52)

$\frac{dT_{PB,4e}}{dt}=\phi\gamma_{4}T_{PB,4n}-k_{V}(1-E_{RT,T_{4}}^{Cell}RT)\frac{V_{PB}}{c_{2}+V_{PB}}T_{PB,4e}-\mu_{4e}T_{PB,4e},$ (53)

(54)

$\frac{dT_{PB,4e}^{i}}{dt}=\phi(1-p)\gamma_{4}T_{PB,4n}^{i}+k_{V}(1-E_{RT,T_{4}}^{Cell}RT)\frac{V_{PB}}{c_{2}+V_{PB}}T_{PB,4e}-(k_{8}\tilde{T}_{PB,8e}+\mu_{4e}^{i})T_{PB,4e}^{i},$ (55)

(56)

(57)

$\frac{dT_{PB,8n}}{dt}=(1-v)(1-f)s+(1-\phi)\gamma_{8}T_{PB,8n}-\mu_{8n}T_{PB,8n},$ (58)

$\frac{dT_{PB,8e}}{dt}=\phi\gamma_{8}T_{PB,8n}-\mu_{8e}T_{PB,8e},$ (59)

(60)

(61)

$\frac{dM_{o}^{a}}{dt}=\frac{V_{PB}}{c_{2}+V_{PB}}(M_{o}-M_{o}^{i}-M_{o}^{a})-\mu_{Mo}^{a}M_{o}^{a}-(P_{LN}+P_{CNS})M_{o}^{a},$ (62)

(63)

(64)

(65)

(66)

(67)

(68)

$\begin{matrix} \frac{dT_{LN,4n}}{dt}=(1-v)f(1-\frac{eV_{LN}}{c_{1}+V_{LN}})s+(1-\phi)\gamma_{4}T_{LN,4n}-k_{V}(1-E_{RT,T_{4}}^{Cell}E_{RT}^{Dept}RT)\frac{V_{LN}}{c_{2}+V_{LN}}T_{LN,4n} \\ \begin{matrix} & & & -\mu_{4n} \end{matrix}T_{LN,4n}-C_{T4}^{Mi}(1-E_{PI}^{Dept}PI)\mu_{4n}M^{i}T_{LN,4n}, \end{matrix}$ (69)

$\frac{dT_{LN,4e}}{dt}=\phi\gamma_{4}T_{LN,4n}-k_{V}(1-E_{RT,T_{4}}^{Cell}E_{RT}^{Dept}RT)\frac{V_{LN}}{c_{2}+V_{LN}}T_{LN,4e}-\mu_{4e}T_{LN,4e},$ (70)

(71)

$\frac{dT_{LN,4e}^{i}}{dt}=\phi(1-p)\gamma_{4}T_{LN,4n}^{i}+k_{V}(1-E_{RT,T_{4}}^{Cell}E_{RT}^{Dept}RT)\frac{V_{LN}}{c_{2}+V_{LN}}T_{LN,4e}-(k_{8}\tilde{T}_{LN,8e}+\mu_{4e}^{i})T_{LN,4e}^{i},$ (72)

(73)

(74)

$\frac{dT_{LN,8n}}{dt}=(1-v)(1-f)s+(1-\phi)\gamma_{8}T_{LN,8n}-\mu_{8n}T_{LN,8n},$ (75)

$\frac{dT_{LN,8e}}{dt}=\phi\gamma_{8}T_{N,8n}-\mu_{8e}T_{LN,8e},$ (76)

(77)

(78)

$\frac{dM^{a}}{dt}=\frac{V_{LN}}{c_{2}+V_{LN}}(M-M^{i}-M^{a})-\mu_{M}^{a}M^{a}+P_{LN}M_{o}^{a},$ (79)

$\frac{dM^{i}}{dt}=k_{V,M}(1-E_{RT}^{Dept}RT)\frac{V_{LN}}{c_{2}+V_{LN}}(M-M^{i})+P_{LN}M_{o}^{i}-\mu_{M}^{i}M^{i}-k_{8}\delta\tilde{T}_{LN,8e}M^{i},$ (80)

(81)

$\frac{dFDC^{i}}{dt}=\overline{k_{V,M}}(1-E_{RT}^{Dept}RT)\frac{V_{LN}}{c_{2}+V_{LN}}(N_{ICs}(1-\frac{t}{D_{FDC^{i}}})-FDC^{i})-\mu_{FDC}^{i}FDC^{i}.$ (82)
